# First finding of free-living representatives of Prokinetoplastina and their nuclear and mitochondrial genomes

**DOI:** 10.1038/s41598-021-82369-z

**Published:** 2021-02-03

**Authors:** Denis V. Tikhonenkov, Ryan M. R. Gawryluk, Alexander P. Mylnikov, Patrick J. Keeling

**Affiliations:** 1 Papanin Institute for Biology of Inland Waters, Russian Academy of Sciences, Borok, 152742 Russia; 2 AquaBioSafe Laboratory, University of Tyumen, 625003 Tyumen, Russia; 3 Department of Biology, University of Victoria, Victoria, British Columbia V8W 2Y2 Canada; 4 Department of Botany, University of British Columbia, Vancouver, British Columbia V6T 1Z4 Canada

**Keywords:** Phylogenetics, Taxonomy, Eukaryote, Evolutionary biology, Genome, Genomics, Cytoskeleton

## Abstract

Kinetoplastids are heterotrophic flagellated protists, including important parasites of humans and animals (trypanosomatids), and ecologically important free-living bacterial consumers (bodonids). Phylogenies have shown that the earliest-branching kinetoplastids are all parasites or obligate endosymbionts, whose highly-derived state makes reconstructing the ancestral state of the group challenging. We have isolated new strains of unusual free-living flagellates that molecular phylogeny shows to be most closely related to endosymbiotic and parasitic *Perkinsela* and *Ichthyobodo* species that, together with unidentified environmental sequences, form the clade at the base of kinetoplastids. These strains are therefore the first described free-living prokinetoplastids, and potentially very informative in understanding the evolution and ancestral states of morphological and molecular characteristics described in other kinetoplastids. Overall, we find that these organisms morphologically and ultrastructurally resemble some free-living bodonids and diplonemids, and possess nuclear genomes with few introns, polycistronic mRNA expression, high coding density, and derived traits shared with other kinetoplastids. Their genetic repertoires are more diverse than the best-studied free-living kinetoplastids, which is likely a reflection of their higher metabolic potential. Mitochondrial RNAs of these new species undergo the most extensive U insertion/deletion editing reported so far, and limited deaminative C-to-U and A-to-I editing, but we find no evidence for mitochondrial *trans*-splicing.

## Introduction

Kinetoplastids constitute a diverse group of free-living and parasitic protists, including animal and human pathogens (e.g., *Trypanosoma* and *Leishmania*), as well as widespread, cosmopolitan, free-living bodonids, which play a major role in marine and freshwater ecosystems as bacterial consumers. Kinetoplastids are members of the phylum Euglenozoa, a subgroup of excavate protists that also includes poorly-studied anaerobic symbiontids^1^, along with euglenids and diplonemids, which, like kinetoplastids, are characterized by unusual nuclear and mitochondrial genomes^2^. Kinetoplastids are named for the kinetoplast^3^—the dense DNA-containing structure now known to be the genome of their single giant mitochondrion, which is associated with the base of the flagella.

To date, there is limited information regarding the origin and evolutionary history of kinetoplastids. Traditional taxonomy divides class Kinetoplastea Honigberg 1963 into two groups—the free-living Bodonina and parasitic Trypanosomatina^4–6^. Modern classification based on molecular data classifies Kinetoplastea into two new and slightly different subclasses: the Prokinetoplastina and Metakinetoplastina, the latter including the parasitic Trypanosomatida as well as three recently added suborders of predominantly free-living bodonids—Eubodonina, Parabodonina, and Neobodonina^7,8^. Multigene phylogenies indicate that bodonids are paraphyletic, making up several lineages, one of which is sister to trypanosomatid parasites^9,10^. Thus, reconstructing kinetoplastid phylogeny and evolution has been intertwined with questions of how parasitism originated within the group.

Prokinetoplastina is currently composed of two genera: the parasitic flagellates, *Ichthyobodo*, and the amoeboid endosymbiont, *Perkinsela*^7,11^. *Ichthyobodo* comprises marine and freshwater fish ectoparasites that infect epithelial cells, causing the often lethal disease Ichthyobodosis, or Costiosis. Their cells are usually pyriform, attach to the host and feed by the use of a cytostome and cytopharyngeal canal, which protrudes into the host cell^12^. Non-feeding free-swimming cells are oval and move using two (rarely four) unequal flagella^13^. All known *Perkinsela* species are obligate endosymbionts of *Neoparamoeba*—itself a fatal gill parasite of fish, including salmonids (amoebic gill disease, AGD). *Perkinsela* lives in the amoeba cytoplasm, in close contact with the nucleus, and the association is considered to be a mutualistic relationship^14^.

Morphological information on *Perkinsela* is very limited but available molecular data indicate that this organism is highly divergent and possesses the smallest kinetoplastid gene repertoire reported so far^15^. *Perkinsela* retains hallmark features of kinetoplastid biology, including polycistronic transcription, *trans*-splicing, and a glycosome-like organelle^15^, but has lost the genes necessary to make flagella, and possesses a derived and reduced metabolic capacity. For instance, *Perkinsela* lacks numerous enzymes, including those required for methionine recycling (methylthioadenosine phosphorylase); the biosynthesis of uric acid, pyrimidines, cysteine, and glutathione; reductive uracil and thymine degradation; and elongases involved in fatty acid biosynthesis^16^. The molecular features of *Perkinsela* are fascinating from the perspective of understanding the fate of eukaryotic endosymbionts^15^, but provide less insight into the ancestral state of kinetoplastids because they are themselves so highly derived from the ancestral state. No genomic and transcriptomic data are currently available for *Ichthyobodo* parasites, but as parasites, they too might be highly derived.

Reconstructing the ancestral state of kinetoplastids would benefit greatly from analysis of less-derived, free-living prokinetoplastid species, but until recently none were known. However, in a recent comparative transcriptomic survey of Euglenozoa we showed that free-living flagellate clones PhF-6 and PhM-4 were related to Prokinetoplastina^16^. Analysis of their transcriptomes showed that they encode a higher number of metabolic proteins than other kinetoplastids, and that their metabolic capabilities are more similar to diplonemids and euglenids^16^. These organisms are clearly of great interest to our understanding of the morphological and molecular characteristics of the ancestor of kinetoplastids, and the evolution of their unusual mitochondrial genomes, including editing of mitochondrial mRNAs.

Here we combined culturing, morphological, ultrastructural, and molecular approaches to characterize these protists and their mitochondrial and nuclear genomes, and also formally describe the strains as new genera and species, *Papus ankaliazontas* n. gen. n. sp. and *Apiculatamorpha spiralis* n. gen. n. sp. As the first free-living representatives of prokinetoplastids to be described, we sought to use these organisms to aid in the reconstruction of ancestral states of kinetoplastids to further our understanding of the evolution of both their morphological and genomic features.

## Methods

### Clone isolation and culture maintenance

Clone PhM-4 (*Papus ankaliazontas* n. gen. n. sp.) was sampled on May 16, 2014 from macrophyte-associated detritus at the shore of a brackish lagoon (~ 8‰) named Lake Küçükçekmece (Istanbul, Turkey). Clones PhF-5 and PhF-6 (*Apiculatamorpha spiralis* n. gen. n. sp.) were isolated from freshwater volcanic sediments of Lake Toba (near Tuc Tuc village, Sumatra, Indonesia) on November 21, 2014, and from macrophyte-associated detritus in a small freshwater lake near Noi Bai International Airport (Hanoi, Vietnam) on May 1, 2015, respectively. Samples were examined on the third, sixth, and ninth days of incubation in accordance with methods described previously^17^.

*Procryptobia sorokini* strain B-69 (IBIW RAS), feeding on *Pseudomonas fluorescens*, was cultivated in Schmaltz-Pratt’s medium at a final salinity of 20‰, and used as prey for clone PhM-4^18^. Freshwater clones PhF-5 and PhF-6 were propagated on *Parabodo caudatus* strain BAS-1 (IBIW RAS) in Pratt’s medium using *Pseudomonas fluorescens* as food. Clone PhM-4 perished after three years of cultivation. The clones PhF-5 and PhF-6 are stored in the collection of live protozoan cultures at IBIW RAS.

### Microscopy

Light microscopy observations were made by using the Zeiss AxioScope A.1 equipped with a DIC contrast water immersion objective (63x). The images were taken with an AVT HORN MC-1009/S analog video camera and directly digitized using the Behold TV 409 FM tuner.

Cells were fixed for transmission electron microscopy (TEM) after centrifugation at 1 °C for 15–60 min in a cocktail of 0.6% glutaraldehyde and 2% OsO_4_ (final concentration) prepared in a 0.1 M cacodylate buffer (pH 7.2) for clones PhF-5 and PhF-6, or diluted Schmaltz-Pratt medium (20 ‰) for strain PhM-4. Fixed cells were dehydrated in an alcohol and acetone series (30, 50, 70, 96, and 100%; 20 min per step). Cells were embedded in a mixture of Araldite and Epon^19^. Ultrathin sections were prepared with an Leica EM UC6 ultramicrotome (Leica Microsystems, Germany) and observed using a JEM 1011 transmission electron microscope (JEOL, Japan).

Scanning electron microscopy (SEM) was performed on cells from exponential growth phase that were fixed as for TEM, but only for 10 min at 22 °C, and gently drawn onto a polycarbonate filter (diameter 24 mm, pores 0.8 µm) as described previously^20^. Following filtration, the specimens were dehydrated through a graded series of ethanol and acetone treatment, and finally put into a chamber of a critical point device for drying, then dry filters with fixed specimens were mounted on aluminum stubs, coated with gold–palladium, and observed with a JSM-6510LV scanning electron microscope (JEOL, Japan)^20^.

### 18 rRNA gene sequencing

The 18S rRNA gene sequence of isolate PhM-4 (GenBank Accession Number MW346654) was amplified using general eukaryotic primers PF1 and FAD4^18^. Products were cloned and sequenced by Sanger dideoxy sequencing. 18S rRNA genes of *A. spiralis* isolates PhF-5 and PhF-6 (GenBank Accession Numbers MW346652 and MW346645) were amplified and sequenced by Sanger dideoxy sequencing with eukaryote-specific primers EukA and EukB^21^ and PF1 and FAD4, respectively.

### Preparation of cDNA and genomic DNA

For cDNA preparation, cells grown in clonal laboratory cultures were harvested following peak abundance, after eating most of the prey, as described previously^22^. Cells were collected by centrifugation (2000×*g*, room temperature) on a 0.8 µm membrane of Vivaclear Mini columns (Sartorius Stedim Biotech Gmng, Germany, Cat. No VK01P042) followed by total RNA extraction using an RNAqueous-Micro Kit (Invitrogen, Cat. No AM1931) and conversion into cDNA prior to sequencing^22^ using the SMARTer Pico PCR cDNA Synthesis Kit (Clontech, lot # 1308018A) for clone PhM-4, and the Smart-Seq2 protocol^23^ for clones PhF-5 and PhF-6. Paired-end libraries were prepared using the NexteraXT protocol (Illumina, Inc., Cat. # FC-131–1024), and sequencing was performed on an Illumina MiSeq platform with read lengths of 2 × 250 bp (PhM-4) and 2 × 300 bp (PhF-5, PhF-6). Additionally, total RNA-seq was performed on an Illumina HiSeq 2500 platform with read lengths of 2 × 100 bp, using the KAPA stranded RNA-seq kit (Roche) to construct paired-end libraries.

Genomic DNA was isolated from all cultures using an Epicentre DNA extraction kit (Cat. No. MC85200) and libraries were prepared using the Nextera library preparation protocol (Illumina, Inc., Cat. #FC-121–1030). In the case of *P. ankaliazontas*, ~ 122,000 target cells were sorted using a FACSAria IIu (BD Biosciences) prior to DNA extraction. Paired-end reads (2 × 300 bp) were sequenced on an Illumina MiSeq instrument.

### Genome assembly

MiSeq paired-end DNA reads for each species were trimmed of adapter and low-quality sequence with bbduk, and overlapping reads were merged with bbmerge using default parameters (bbmap version 37.06). Merged and unmerged paired-end reads were assembled using SPAdes version 3.11.1^24^ with kmer lengths of 21, 33, 55, 77, 99 and 121. For assembly with Ray v2.3.1^25^, a kmer value of 107 was used. Ray assemblies were used only for aiding in interpretation of mitochondrial genome datasets; SPAdes was used for all nuclear genomic analyses. For *A. spiralis*, we generated separate assemblies for PhF-5 and PhF-6, used in examining mitochondrial genomes and RNA-editing, along with a combined assembly for evaluating the nuclear genome. Genomic contamination from prokaryotes was identified using Autometa^26^, analyszing only contigs greater than 1500 bp. Contaminating genomic sequences from eukaryotic prey were identified with megablast using either prey transcriptomes as query, or transcriptomes from other projects that included the same prey species; contaminants were defined empirically as contigs with ≥ 93% identity to contigs from the respective prey transcriptome datasets over a length of ≥ 100 bp. Genome assembly quality was evaluated with QUAST v5.0.2^27^. Estimates of nuclear genome size were performed with two different software packages on reads that map to the decontaminated genome assemblies. Initially, kmerfreq^28^ was used to determine kmer frequency, employing a kmer length of 17, and the Bayesian model program GCE^28^ used the kmer frequencies to predict genome size, under the ‘gce-alternative’ framework. Additionally, kmercountexact.sh from the bbmap package was used, employing default parameters (k = 31).

*P. ankaliazontas* and *A. spiralis* (strain PhF-6) RNA-seq reads were trimmed, assembled and decontaminated as recently described^16^. Transcripts ≥ 95% identical were clustered with cd-hit^29^, and coding sequences were predicted using Transdecoder v5.3.0^30^. Predicted coding sequences were mapped to genomic contigs with blat v36 × 1^31^ requiring a minimum 90% sequence identity, to search for spliceosomal introns and assess gene polarity and clustering, and isoblat v0.3^32^ was used to determine the proportion of transcripts that map to genomic contigs.

### Mitochondrial genome and RNA-editing analysis

*P. ankaliazontas, A. spiralis*, and prey kinetoplastid mitochondrial protein-coding genes were identified by searching transcriptome assemblies with sequences of proteins typically encoded in mtDNA, using tblastn or hmmer^33^. Putative mitochondrial rRNA genes were identified with scan_for_matches^34^ by searching the genome and transcriptome assemblies for motifs that are well conserved in the *Leishmania tarentolae* 9S (530 loop) and 12S (A-loop) rRNAs.

Mitochondrial transcripts were used to identify likely mitochondrial genome contigs (*i.e.*, maxicircles) and guide RNA-encoding contigs (*i.e.*, minicircles) using blastn, with a reduced word size of 4. Where mitochondrial mRNAs were pan-edited, and corresponding gene sequences were impossible to identify using standard blastn, we deleted all thymidines from the query and searched a database similarly depleted of thymidine, with the ‘-dust no’ and ‘-soft_masking false’ flags activated. Mitochondrial RNA-editing was reconstructed by manually aligning transcripts to maxicircle gene regions, assuming frequent U indels, as found in other kinetoplastids.

To investigate whether *P. ankaliazontas* mtDNA contigs are circular-mapping, we used a seed-based approach with NOVOPlasty v3.7.1^35^, using a kmer length of 39. The depth of coverage of *A. spiralis* PhF-5 and PhF-6 maxicircles was insufficient for this approach, regardless of kmer length.

### Phylogenetic reconstructions

Small subunit ribosomal RNA gene sequences were manually selected from diverse diplonemids, and kinetoplastids, with the aim of including nearly full-length sequences from a diverse species range. Sequences were aligned using MAFFT v7.212^36^ and trimmed with trimAl v1.4^37^ with a gap threshold (gt) of 0.3 and a minimum average similarity (st) of 0.001. Phylogenetic trees were reconstructed using IQ-TREE v1.6.12^38^ under the GTR + F + I + G4 model with 200 initial parsimony trees and 1000 ultrafast bootstrap replicates. Additional 18S gene trees were rooted with euglenids, and symbiontids, but were otherwise reconstructed identically.

Phylogenetic trees were generated for mitochondrial proteins with IQ-TREE using the LG + G4 + Γ model, including correction for absence of invariant sites and 100 non-parametric bootstrap replicates as measures of statistical support.

## Results

### Phylogenetic analysis of the 18S rRNA gene

**Figure 1:**
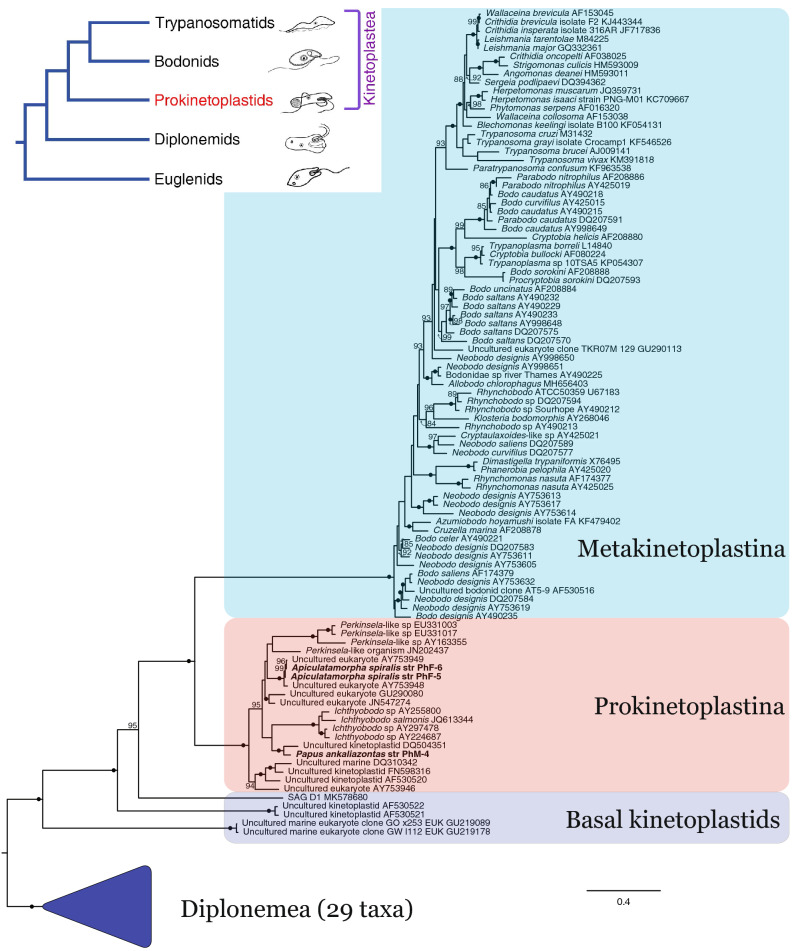
*P. ankaliazontas* and *A. spiralis* are prokinetoplastids. A maximum likelihood phylogeny of the 18S ribosomal RNA gene from diverse kinetoplastids and diplonemids (outgroup) was reconstructed with IQ-TREE v1.6.12 using the GTR + F + I + G4 model with 200 initial parsimony trees and 1000 ultrafast bootstrap replicates as a measure of support. *P. ankaliazontas* and *A. spiralis* affiliate with strong support with Prokinetoplastina, yet their precise relationships to parasitic and endosymbiotic prokinetoplastid groups could not be resolved. For clarity, ultrafast bootstrap values less than 85 are not shown. A phylogeny including more diverse euglenozoans as outgroup is presented in Supplementary Material, Fig. S1.

Phylogenetic analysis of the 18S ribosomal RNA gene places both *P. ankaliazontas* and *A. spiralis* within Prokinetoplastina, a fully supported clade nested between a small number of environmental ‘basal’ kinetoplastid sequences, and metakinetoplastids (Fig. 1). Within Prokinetoplastina, *P. ankaliazontas* is placed with full bootstrap support as sister to an environmental sequence isolated from the ‘Lost City’ deep sea hydrothermal field^39^. In turn, both are sister to a clade dominated by parasitic *Ichthyobodo* spp., but with moderate support (74% bootstrap support). *A. spiralis* strains PhF-5 and PhF-6 18S sequences (99% identical to each other) branch with freshwater environmental sequences from Panama and Botswana (99% bootstrap support)^40^. The phylogeny recovers *A. spiralis* and close allies as sister to endosymbiotic *Perkinsela*-like organisms, but this relationship is not statistically robust, is not recovered in similar phylogenetic trees employing euglenids and symbiontids as the outgroup (Supplementary Material, Fig. S1), and is inconsistent with a recent multigene phylogeny that recovered *A. spiralis* and *P. ankaliazontas* as close relatives^16^.

### External morphology and ultrastructure

#### *Papus ankaliazontas*, clone PhM-4

**Figure 2:**
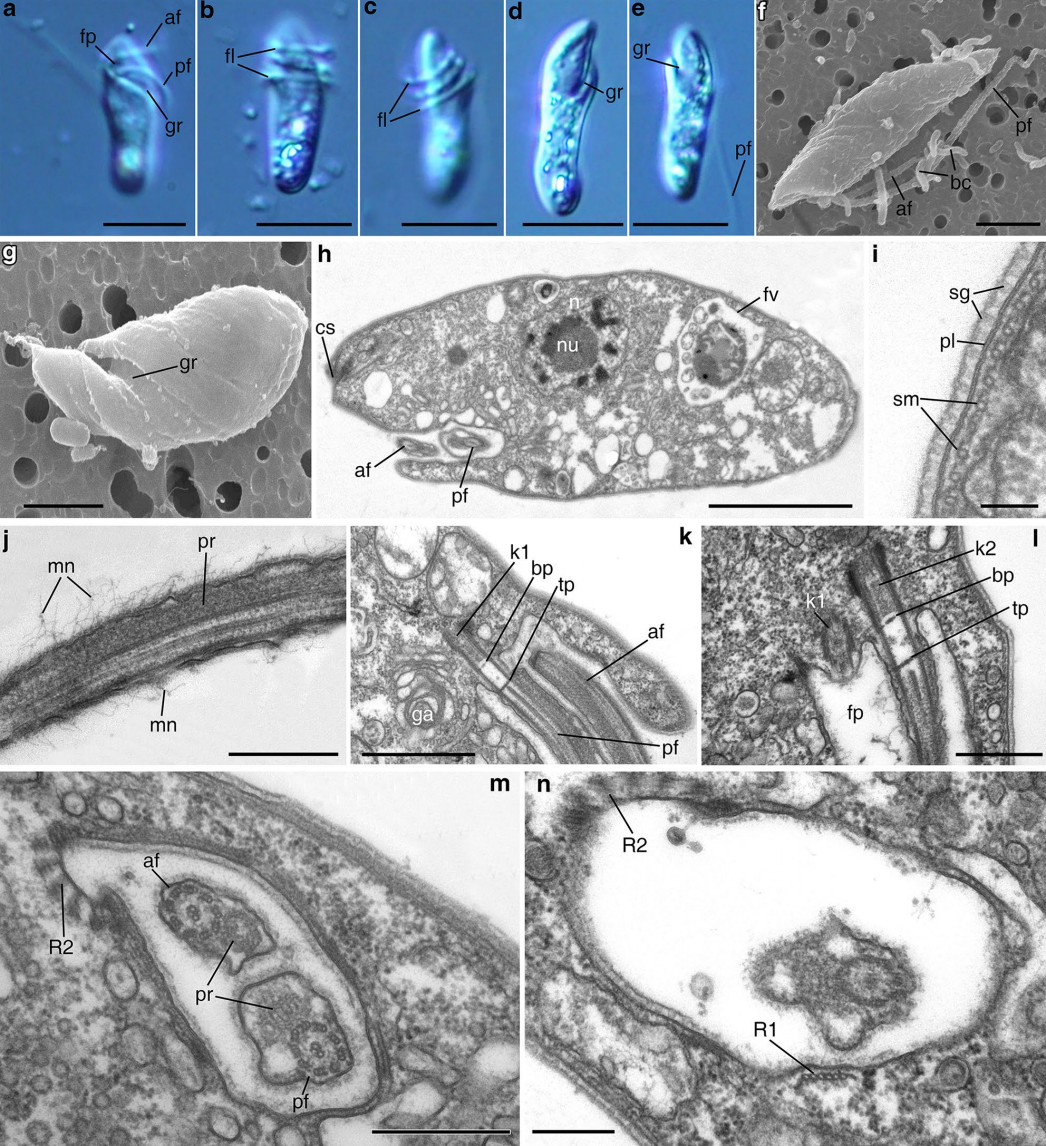
General view, cell coverings, and flagellar pocket of *Papus ankaliazontas*, clone PhM-4. **(a–e)** living cells, LM, DIC; **(f, g)** general cell view, SEM (**g** flagella are not visible); **(h)** longitudinal cell section; **(i)** coverings structure; **(j)** anterior flagellum; **(k, l)** arising of the flagella from flagellar pocket; **(m, n)** cross sections of the flagellar pocket. *af *anterior flagellum, *bc* bacterium, *bp* basal plate, *cs* cytostome, *fl* flagella, *fp* flagellar pocket, *fv* food vacuole, *ga* Golgi apparatus, *gr* groove, *k1* kinetosome (basal body) of the posterior flagellum; *k2* kinetosome (basal body) of the anterior flagellum, *mn* mastigonemes, *n* nucleus, *nu* nucleolus, *pf* posterior flagellum, *pl* plasmalemma, *pr* paraflagellar rod, *R1* mucrotubular root R1, *R2* mucrotubular root R2, *sg* structured glycocalyx, *sm* subpellicular microtubules, *tp* transversal plate. Scales: (**a**–**e**) 10, (**f**) 3, (**g**) 2, (**h**) 3, (**i**) 0.1, (**j**) 0.5, (**k**) 1, (**l**–**m**) 0.5, (**n**) 2.5 μm.

A general cell view is presented in Fig. 2 a–g. Cells are rigid, elongated cylindrical, 15–20 μm long, with a roundish posterior end and a slightly asymmetrically located anterior rostrum. A very deep flagellar pocket is situated below the rostrum (Fig. 2a). Two heterodynamic flagella originate from the flagellar pocket and do not adhere to each other. The anterior flagellum is slightly shorter than the cell body, and the posterior flagellum is two times longer than the cell. Flagellates swim rapidly and rotate around their longitudinal axis. The two flagella often wrap spirally around the anterior part of the body, apparently causing the cell to stop (Fig. 2b,c).

The pronounced groove starts ventrally from the flagellar pocket and turns up to the dorsal side of the cell (Fig. 2d,e,g). The cytostome is situated apically above the flagellar pocket (Fig. 2 h). The organism is a predator, and captures prey entirely, feeding on other flagellates (e.g. the bodonid *Procryptobia sorokini* in culture) and perishes in the absence of eukaryotic prey. Cells multiply by binary longitudinal division. Cysts have not been found in culture.

The main cell structures are visible in the longitudinal section (Fig. 2h). The cell is covered with a dense, thick (30–35 nm) layer of structured glycocalyx (Fig. 2i). The glycocalyx is absent in the bottom part of the flagellar pocket (Fig. 2l). A system of subpellicular microtubules (corset) connected by crosspieces lays beneath a plasmalemma (Fig. 2i). The intervals between the centers of microtubules are about 42–50 nm. Distinct gaps in the corset were not observed. Most of the microtubules have a longitudinal or slightly oblique orientation.

Two flagella originate almost in parallel from the bottom of the flagellar pocket (Fig. 2k,l). The anterior flagellum is covered by thin crimp hairs (mastigonemes) (Fig. 2j). Kinetosomes (basal bodies) are long and have typical structure (Fig. 2k,l). Two central microtubules of the flagellar axoneme originate close to the transverse terminal plate (Fig. 2k). The basal plate resembles a flattened ring (Fig. 2k,l). Both flagella have paraflagellar rods, which start at the level of the terminal plate in the flagellar transition zone and face each other (Fig. 2k,m). The paraflagellar rod of the posterior flagellum has reticulate structure, whereas the paraflagellar rod of the anterior flagellum is less developed and is characterized by a ring-shaped structure in the cross section (Fig. 2m).

**Figure 3:**
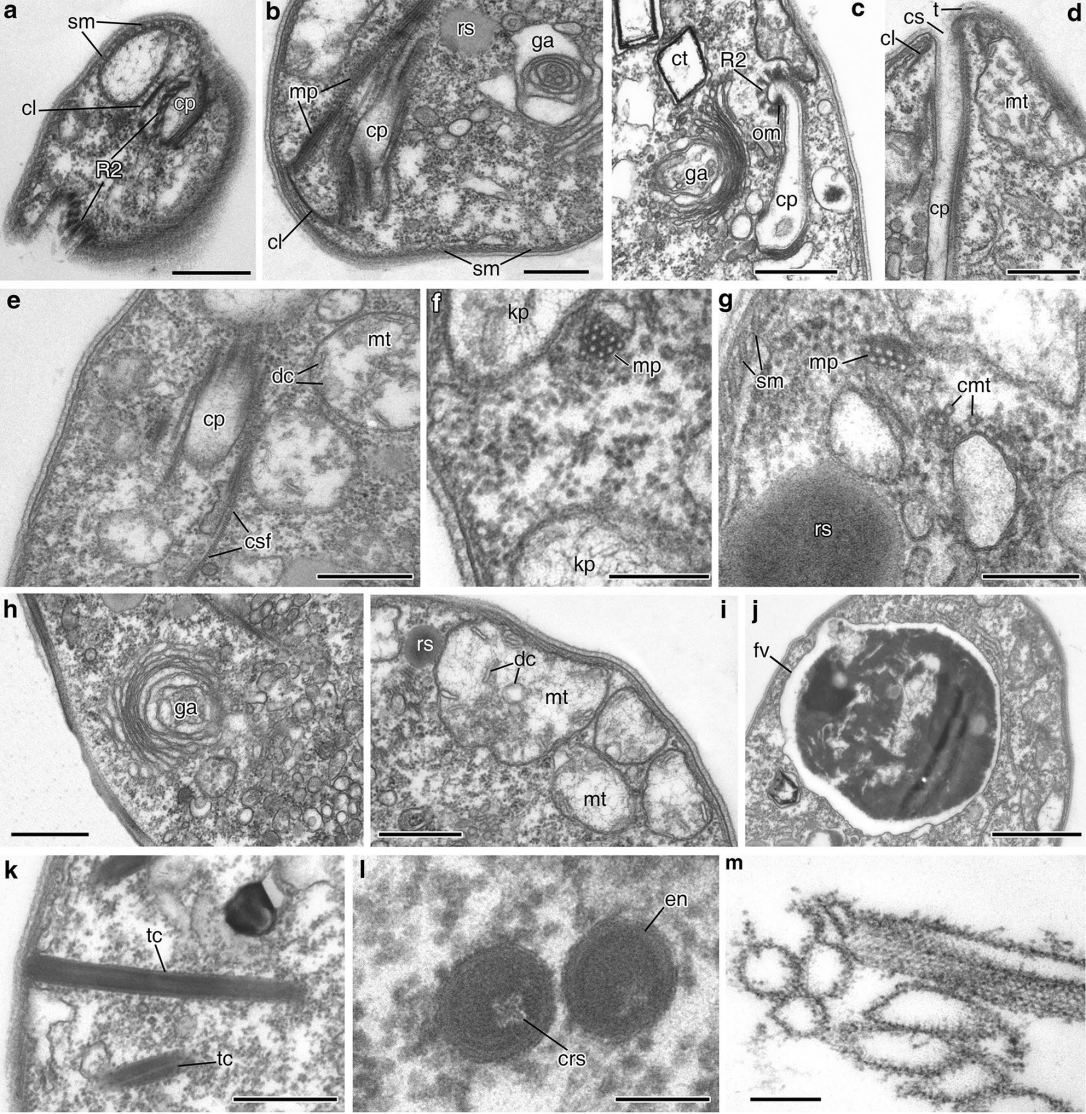
Structure of the anterior cell end, cytopharynx, and cell organelles of *Papus ankaliazontas*, clone PhM-4. **(a–c)** cross sections of the apical cell part; **(d)** longitudinal section of cytopharynx; **(e)** cytopharynx reinforced by cross-striated fibril; **(f,g)** microtubular prism; **(h)** Golgi apparatus; **(i)** mitochondria; **(j)** food vacuole; **(k)** longitudinal section of the trichocysts; **(l)** cross section of the trichocysts with cruciform structure; **(m)** empty envelopes of the trichocysts after discharging. *cl* clamp, *cmt* cytopharynx associated additional microtubules, *cp* cytopharynx, *cs* cytostome, *crs* cruciform structure, *csf* cross-striated fibril, *ct* crystalloid structure, *dc* discoid cristae, *en* envelope of the trichocyst, *fv* food vacuole, *ga* Golgi apparatus, *kp* kinetoplast, *mp* microtubular prism, *mt* mitochondrion, *om* osmiophilic material, *R2* mucrotubular root R2, *rs* reserve substance, *sm* subpellicular microtubules, *t* tomentum, *tc* trichocyst. Scales: (**a**–**d**) 0.5, (**e**) 0.5, (**f**,**g**) 0.2, (**h**,**i**) 0.5, (**j**) 1, (**k**) 0.5, (**l**) 0.1, (**m**) 0.2 μm.

Two microtubular roots, R1 and R2, were observed in cross sections of the flagellar pocket area (Fig. 2m,n). A third dorsal root, R3, was not visualized but is assumed to be present. The most remarkable root, R2, consists of 5–6 microtubules (Fig. 2m,n); this root passes towards the anterior cell end, bends and continues under the membrane of the cytopharynx along the entire cytopharynx length (Fig. 3a,c). The cytopharynx appears in sections as a tube (Fig. 3d) reinforced by several structures: root R2, a microtubular prism, a cross-striated fibril, and an apical osmiophilic clamp (Fig. 3a,b,e). The cytopharynx is long and covered by a tomentum (fringe of thin hairs) in the proximal part (Fig. 3d). Microtubules of R2 are connected with the cytopharynx by osmiophilic material (Fig. 3c). Three additional cytopharynx-associated microtubules (CMT) are visible near microtubular root R2 (Fig. 3g). A trapezoidal microtubular prism (nemadesm) lies near the cytopharynx and consists of four closely situated rows of microtubules (5 + 4 + 3 + 1 arrangement) anteriorly, and of two rows (5 + 4 arrangement) close to the posterior cell end (Fig. 3e,g). Microtubular root R1 consists of 6 microtubules (Fig. 2n), passes ventrally, and probably embeds in the system of subpellicular microtubules (not shown).

The Golgi apparatus is situated in the anterior end of the cell and is represented by a single dictyosome 0.78–0.88 μm in diameter (Figs. 2k, 3b,c,h). The contractile vacuole is absent. The nucleus is 2.0–2.2 μm in diameter with a central nucleolus, situated near the cell centre (Fig. 2h). Several mitochondria were observed to have discoid cristae (Fig. 3e,i). A network of DNA is visible inside the mitochondria and most probably corresponds to small kinetoplasts (Fig. 3f). Basal bodies and mitochondria lack any connection. The food vacuoles contain engulfed prey (Fig. 3j).

Extrusive organelles (trichocysts) were observed in the cytoplasm (Fig. 3k). Trichocysts are 1.3 μm long, cylindrical in cross section, and 130 nm in diameter. An osmiophilic envelope of the trichocyst surrounds the inner cylinder. The cruciform structure is visible inside the inner cylinder (Fig. 3l). After discharging, the size of the trichocyst envelope is increased to 200 nm, and it becomes reticulate (Fig. 3m). The size of the mesh side is 20–21 nm.

The mesh ribs are formed by two systems of fibrils. The first system consists of fibrils running parallel to the axis of trichocyst, while the second one runs from the fibrils lying at about 120° angle to the axis of trichocyst.

Crystalloid structures (~ 320 × 380 nm) were found in the cytoplasm (Fig. 3c). A storage compound is probably represented by roundish osmiophilic bodies 0.35–0.40 μm in diameter (Fig. 3b,g).

#### *Apiculatamorpha spiralis*, clones PhF-5 and PhF-6

**Figure 4:**
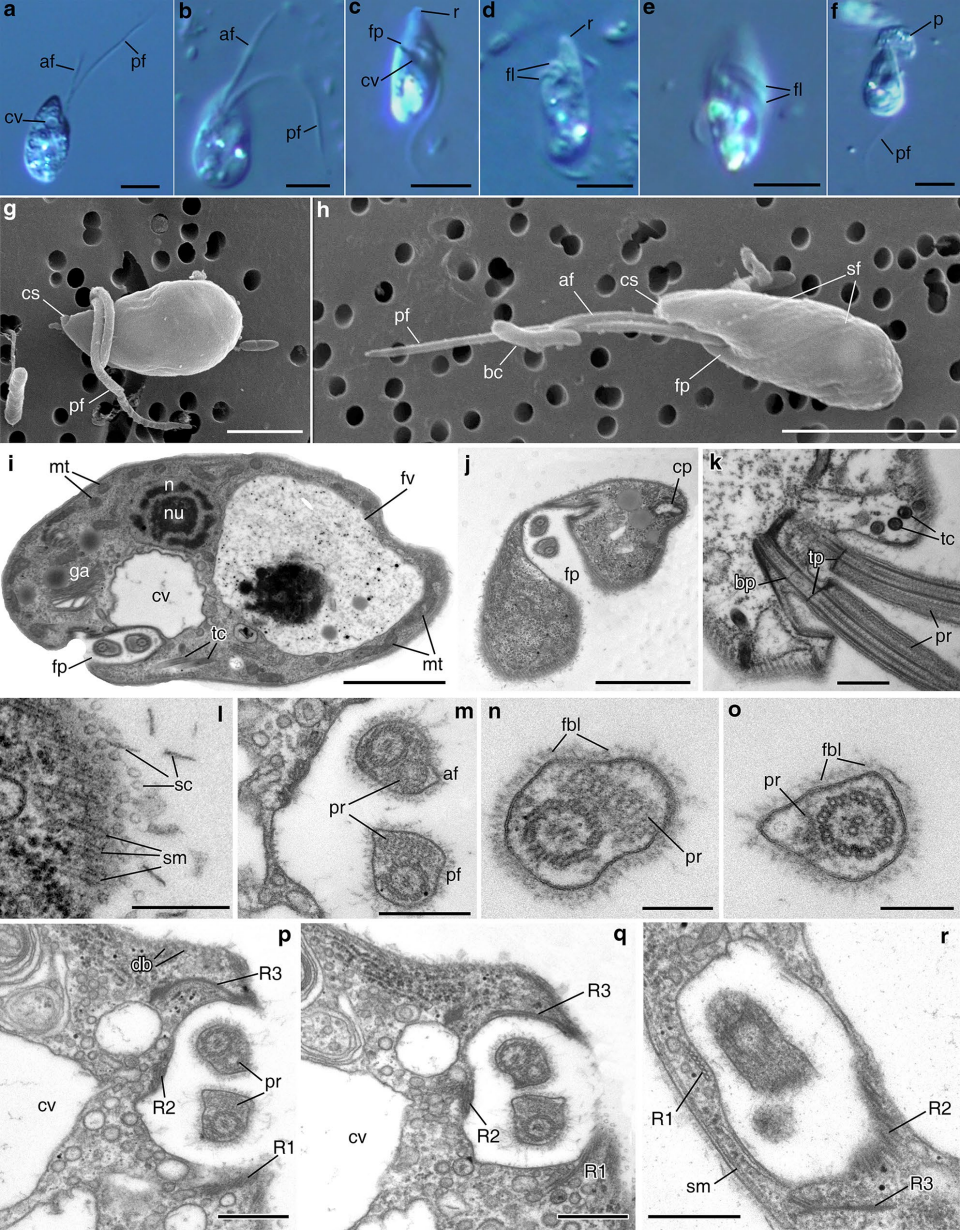
General view, cell coverings and arrangement of flagellar pocket of *Apiculatamorpha spiralis* (**a**–**j**,**l**–**r**—clone PhF-6, **k**—clone PhF-5). **(a–f)** living cell, LM, DIC; **(g,h)** general cell view, SEM; **(i)** longitudinal section of the cell, **(j)** transverse section of the anterior cell end; **(k)** arising of the flagella from flagellar pocket; **(l)** scales on the cell surface; **(m–o)** cross sections of the flagella (**n**—posterior flagellum, **o**—anterior flagellum); **(p–r)** microtubular roots and flagellar pocket. *af* anterior flagellum, *bc* bacterium, *bp* basal plate, *cp* cytopharynx, *cs* cytostome, *cv* contractile vacuole, *db* dorsal band of the microtubules, *fbl* fiber layer, *fl* flagella, *fp* flagellar pocket, *fv* food vacuole, *mt* mitochondrion, *n* nucleus, *nu* nucleolus, *pf* posterior flagellum, *p* prey, *pr* paraflagellar rod, *r* rostrum, *R1* microtubular root R1, *R2* microtubular root R2, *R3* microtubular root R3, *sf* spiral furrow, *sc* scales, *sm* subpellicular microtubules, *tc* trichocyst, *tp* transversal plate. Scales: (**a**–**f**) 5, (**g**) 2, (**h**) 5, (**i**,**j**) 2, (**k**) 0.5, (**l**) 0.2, (**m**) 0.5, (**n**,**o**) 0.2, (**p**–**r**) 0.5 μm.

Clones were morphologically identical. Cells are elongate and oval, elongate oviform, or pear-shaped, 7–13.5 μm long (Fig. 4a–f). A pronounced rostrum is located anteriorly (Fig. 4c,d), and the posterior cell end is roundish. A cytostome is situated at the top of the rostrum (Fig. 4g,h); a very deep flagellar pocket is situated subapically below the rostrum (Fig. 4h). Two heterodynamic flagella originated from the flagellar pocket (Fig. 4c,h), and flagella do not adhere to each other. The anterior flagellum is approximately equal to the cell length, and the posterior flagellum is two times longer than the cell. Both flagella often wrap spirally around the anterior part of the body, apparently causing the cell to stop (Fig. 4d,e). Flagellates swim rapdily and rotate around their longitudinal axis. In contrast with *Papus ankaliazontas*, the flagellar pocket is not continuous with the skewed grove. Some cells have spiral folds across the cell body surface in SEM preparations (Fig. 4h), but these are not visible in TEM sections. Organisms are predators, capturing the prey entirely and feeding on other flagellates (Fig. 4f), *e.g.* the bodonid *Parabodo caudatus* in culture, and perish in the absence of eukaryotic prey. Cells multiply by binary longitudinal division. Cysts have not been found in culture.

**Figure 5:**
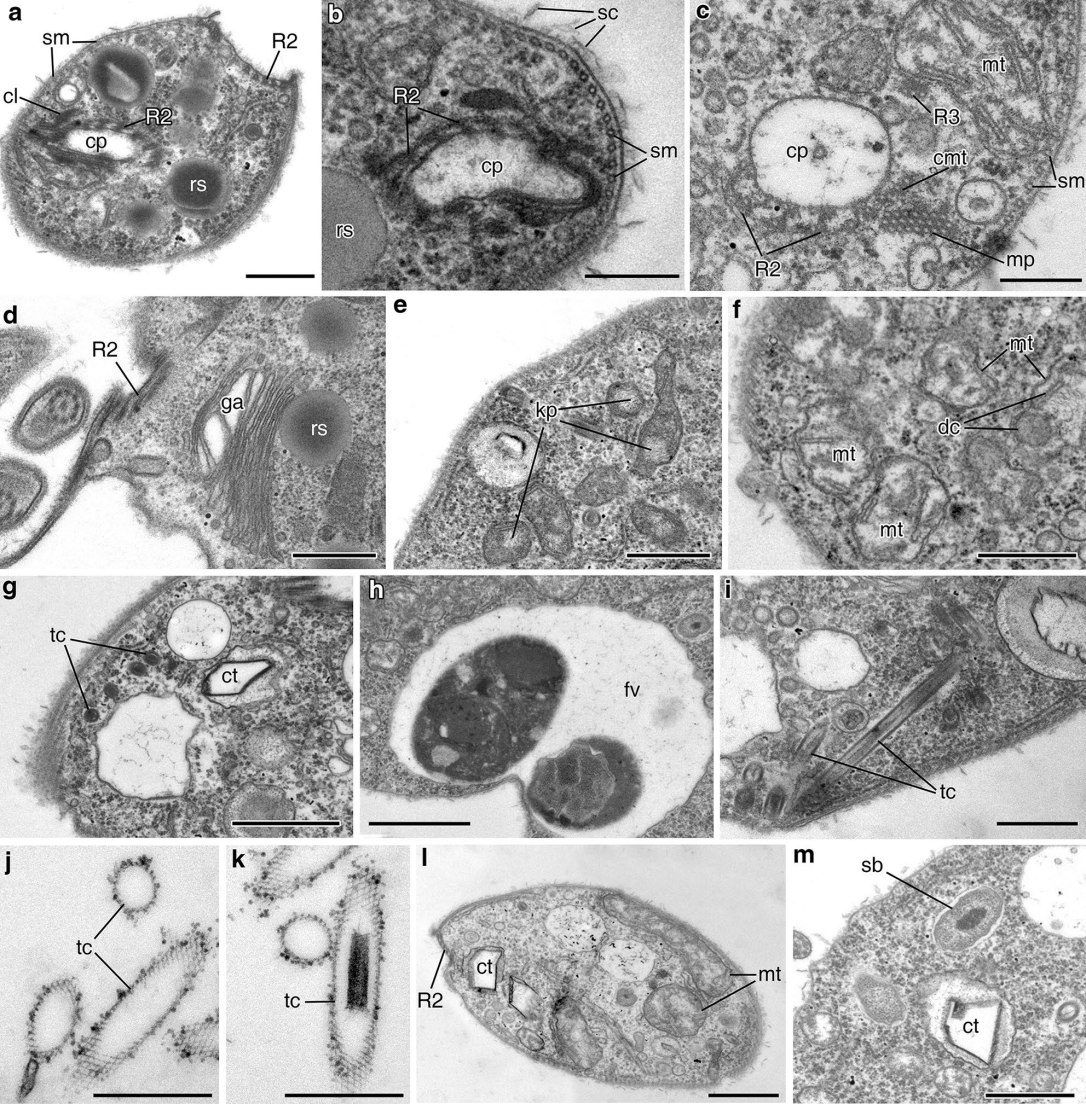
Arrangement of cytopharynx and cell structures of *Apiculatamorpha spiralis*, clone PhF-6, including trichocysts. **(a)** section of the anterior cell end with cytopharynx; **(b, c)** cytopharynx and accociated structures; **(d)** Golgi apparatus; **(e, f)** mitochondria and kinetoplasts; **(g)** trichocysts and crystalloid structure; **(h)** food vacuole; **(i)** longitudinal section of the trichocysts; **(j, k)** empty envelopes of the trichocysts after discharging; **(l)** crystalloid structure and mitochondria; **(m)** symbiotic bacterium and crystalloid structure. *cl* clamp, *cmt* cytopharynx associated additional microtubules, *ct* crystalloid structure, *cp* cytopharynx, *dc* discoid cristae, *fv* food vacuole, *ga* Golgi apparatus, *kp* kinetoplast, *mt* mitochondrion, *mp* microtubular prism, *R2* microtubular root R2, *R3* microtubular root R3, *rs* reserve substance, *sm* subpellicular microtubules, *sb* symbiotic bacterium, *sc* scales, *tc* trichocyst. Scales: (**a**) 0.5, (**b**,**c**) 0.2, (**d**–**f**) 0.5, (**g**,**h**) 1, (**i**–**k**) 0.5, (**l**) 1, (**m**) 0.5 μm.

The longitudinal section of the cell is presented in Fig. 4i, and a transverse section of the apical cell part in Fig. 4j. Two flagella in are present in the flagellar pocket; a cytopharynx, Golgi apparatus, nucleus with nucleolus, contractile vacuole, food vacuole, mitochondria, and trichocysts are visible. The cell is covered with a layer of spherical and lamellate scales (Figs. 4l, 5b); this layer is absent or reduced on the wall of flagellar pocket (Fig. 4i,m). A system of subpellicular microtubules connected by crosspieces lays beneath the plasmalemma (Figs. 4l, r, 5a–c). The intervals between the centers of microtubules are about 30–45 nm. Most of these microtubules have a longitudinal or slightly oblique orientation.

Both flagella have paraflagellar rods. The paraflagellar rod of the posterior flagellum is 0.28 μm wide and reticulate in cross section, while the paraflagellar rod of the anterior flagellum is ring-shaped (Fig. 4 m–q). Paraflagellar rods face each other (Fig. 4k,m). Both flagella are covered with a layer of fine fibers (Fig. 4n,o). Two central microtubules of the flagellar axoneme originate close to the transversal terminal plate; the basal plate appears as a flattened ring (Fig. 4k).

Three microtubular roots originate from kinetosomes (basal bodies). The ventral root, R1, consists of 6 microtubules, the dorsal root, R3, consists of 3 microtubules, and the root, R2, consists of 5–6 microtubules (Figs. 4p–r, 5a,c). Root R3 gives rise to a dorsal band of microtubules (Fig. 4p). Root R1 embeds into the system (corset) of subpellicular microtubules (not shown). Root R2 runs along the wall of flagellar pocket. At the anterior end of the body, it bends backwards and passes under the cytopharyngeal membrane, accompanying the cytopharynx along its entire course (Fig. 5a–c).

The cytopharynx appears in sections as a tube reinforced by several structures: a clamp (in the proximal part), microtubular root R2, a band of three additional cytopharynx associated microtubules (CMT), and the microtubular prism (Fig. 5a–c). The microtubular prism consists of three closely situated rows of microtubules (7 + 8 + 7 arrangement) (Fig. 5c). Microtubules of root R2 are connected with the cytopharynx envelope by osmiophilic material (Fig. 5b).

The nucleus is 1.9 μm in diameter, with a central nucleolus situated slightly closer to the anterior part of the cell (Fig. 4i). The Golgi apparatus is represented by a single dictyosome and is situated in the anterior end of the cell (Figs. 4i, 5d). The contractile vacuole lies near the flagellar pocket (Fig. 4i,p,q). Several small mitochondria possess discoid cristae (Fig. 5c,f,l) and contain kinetoplasts (Fig. 5e). Basal bodies and mitochondria are not visibly connected. The remnants of prey are visible inside food vacuoles (Figs. 4i, 5h).

Crystalloid structures (~ 300 × 450–500 nm) were found in the cytoplasm (Fig. 5g,l,m). Storage compounds are most likely represented by roundish osmiophilic bodies ~ 400 nm in diameter (Fig. 5a,b,d).

Irregular rows of extrusive organelles (trichocysts) lie at the anterior part of the cell (Figs. 4k; 5g,i). Trichocysts are about 1.7 μm long and are cylindrical in cross section. After discharging, the size of the trichocyst envelope is increased, and it becomes reticulate (Fig. 5j,k). The mesh ribs are formed by two systems of fibrils. The first system consists of fibrils running parallel to the axis of trichocyst, while the second one consists of fibrils lying at about a 120° angle to the axis of trichocyst.

Intact symbiotic bacteria were found in the cytoplasm (Fig. 5m).

### Nuclear genome characteristics

Assembly of strand-specific RNA-seq reads generated 42,049 and 64,325 contigs for *P. ankaliazontas* and *A. spiralis*, respectively, after prey decontamination. After clustering transcripts at ≥ 95% identity, Transdecoder^30^, predicted a total of 21,181 and 32,854 ‘genes’ for *P. ankaliazontas* and *A. spiralis*, respectively. Analysis of single-copy ortholog presence indicates that the *P. ankaliazontas* and *A. spiralis* gene repertoires are somewhat larger than other kinetoplastids, which is reflected in their higher metabolic potential^16^.

Assembly of *P. ankaliazontas* and *A. spiralis* genomic reads yielded 22.5 and 24.3 Mbp of assembled sequence, respectively, after decontamination of prey and bacteria (Supplementary Material, Table S1). The *A. spiralis* genome assembly consists of 9,927 contigs larger than 1.5 kbp, with an N50 of 2438 bp. There are 5,118 contigs larger than 1.5 kbp in the *P. ankaliazontas* assembly, and the N50 is 5,464. *P. ankaliazontas* genomic contigs have a higher proportion of G+C (55.8%) than those of *A. spiralis* (48%). Transcript mapping analysis demonstrated at least partial mapping of 73% of *P. ankaliazontas* transcripts to the genome assembly, but only 37% of *A. spiralis* transcripts, indicating that the nuclear genome surveys are quite incomplete. Bayesian kmer frequency approaches using GCE^28^ suggest that the *P. ankaliazontas* and *A. spiralis* nuclear genome sizes are 46 and 70 Mbp. Alternatively, the bbmap package yielded an estimate of 54 Mbp for *P. ankaliazontas*, but could not provide an estimation in *A. spiralis*.

No introns were identified in *P. ankaliazontas* or *A. spiralis* genome surveys. A low number of spliceosomal introns is consistent with their paucity within Kinetoplastida in general, aside from spliced-leader (SL) RNA introns. As observed in other kinetoplastids, the spliceosomal protein repertoire in *P. ankaliazontas* and *A. spiralis* is highly reduced in comparison with other eukaryotes. Some conserved proteins that are absent from *Perkinsela*^15^, including SmD3, SmF, Sm16.5 k, LSm4, LSm7, and U5-40 k, were found in at least one of *P. ankaliazontas* or *A. spiralis*; however, only LSm4 was found in both.

**Figure 6:**
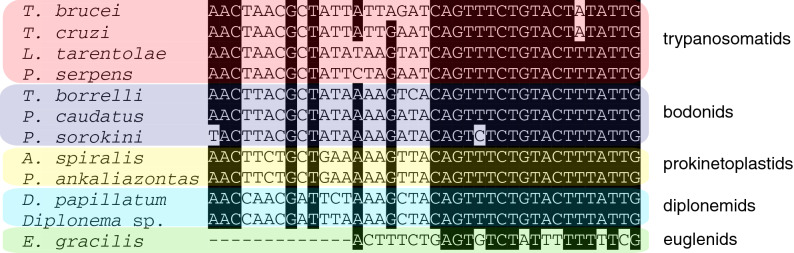
Most prokinetoplastid transcripts contain a spliced-leader (SL) motif similar to those found in other euglenozoans. These sequences are similar to those of SLs from a wide variety of euglenozoans, but are also distinct. The distinctions help to confidently differentiate *bona fide* prokinetoplastid mRNAs from those of their prey, the bodonids *Procryptobia sorokini* and *Parabodo caudatus*. Background shading indicates ≥ 90% sequence identity.

Identical SL-RNA sequences were identified in 70% of *P. ankaliazontas*, and 58% of *A. spiralis* PhF-6 assembled transcripts. These SL-RNA sequences are similar to, but distinct from their counterparts in other euglenozoans (Fig. 6). In *P. ankaliazontas* genomic assemblies, there are ~ 80 contigs that contain a partial version of the 10 bp prokinetoplastid-specific sequence ‘TTACAGTTTCTGTACTT’ (Fig. 6), but only one contains the full-length sequence. This small contig (314 bp) is characterized by a high depth of coverage, and the same contig also encodes a copy of the 5S rRNA gene, suggesting that the SL RNA and 5S rRNA genes are encoded in tandem repeats, as they are in other euglenozoans^41^. In *A. spiralis*, 5 of the 15 contigs containing the 10 bp prokinetoplastid SL sequence are full-length, but none could be linked with the 5S rRNA gene.

Another prominent feature of kinetoplastid genomes is biased gene polarity and clustering. Mapping of strand-specific RNA-seq transcripts to the *P. ankaliazontas* genome assembly confirms that clusters of nuclear genes are transcribed in the same orientation. For instance, the second longest genomic contig (31,686 bp) encodes 15 distinct RNAs, separated on average by 76 bp. Of those, all 15 are transcribed from the same strand. The *A. spiralis* genomic assembly is more fragmentary, but of the 13 RNAs mapping to the longest contig (25,801 bp), all are transcribed in the same orientation, with intergenic distances averaging ~ 1200 bp. Notably, the entire contig is covered by a single 29,462 bp assembled transcript, suggesting polycistronic transcription occurs in *A. spiralis*.

### Mitochondrial genome and RNA-editing

**Figure 7:**
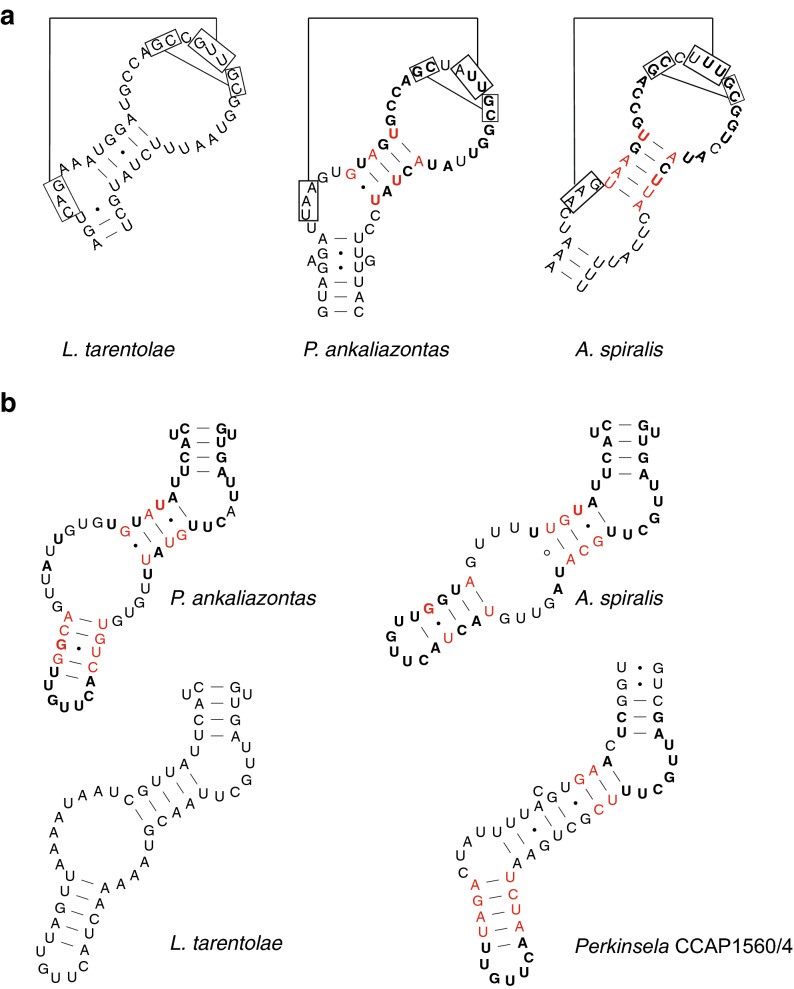
Prokinetoplastid mitochondrial DNA encodes divergent homologs of trypanosomatid 9S and 12S rRNAs. **(a)** Pattern searches for the broadly conserved ‘530′ loop of the small subunit rRNA gene recovered expressed homologs of the 9S rRNA gene from *P. ankaliazontas* and *A. spiralis*. **(b)** Expressed transcripts containing the conserved ‘A-loop’ stem-loop motif of the large subunit rRNA gene (12S) were also found in *P. ankaliazontas, A. spiralis* and possibly *Perkinsela*. Bolded and red nucleotides denote identity and compensatory base changes between prokinetoplastid and *L. tarentolae* homologs, respectively. Boxes in (**a**) highlight complementary nucleotides known to participate in longer range interactions with each other.

In *P. ankaliazontas,* we have identified 14 mtDNA-encoded proteins, including components of respiratory Complexes I (Nad1, Nad4, Nad5, Nad6, Nad7, Nad8, Nad9), III (Cob), IV (Cox1, Cox2, Cox3), V (Atp6), along with the mitochondrial ribosome (Rps12), and a conserved orphan, Murf1 (Supplementary Material, Table S2). A nearly identical gene repertoire was found in *A. spiralis*, though Rps12 and Murf1 could not be identified. Phylogenetic reconstructions of mtDNA-encoded proteins, including Cox1, Cox2, Cox3, and Cob, demonstrate that the *P. ankaliazontas* and *A. spiralis* sequences are basal to bodonids, and do not derive from their prey organisms (Supplementary Material, Fig. S2). Putative homologs of portions of the 9S and 12S rRNA genes were identified in *P. ankaliazontas* and *A. spiralis* transcriptome and mitochondrial genome assemblies by targeted pattern searches for conserved stem-loop structures. Secondary structure predictions of the 530 (9S) and A-loop (12S) motifs demonstrate sequence similarity and compensatory base changes to the corresponding regions of *Leishmania tarentolae* mitochondrial rRNAs (Fig. 7).

**Figure 8:**
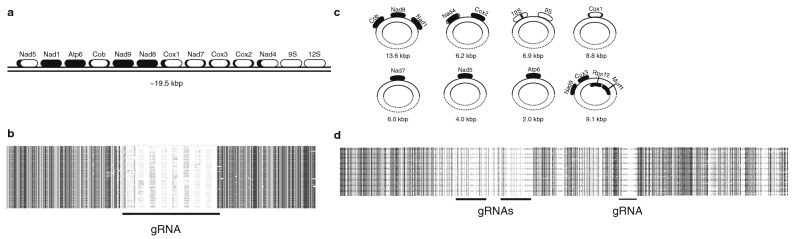
Hypotheses for the structure and content of *A. spiralis* and *P. ankaliazontas* mitochondrial DNA. **(a)** *A. spiralis* ‘maxicircle’ DNA is likely linear, and approximately 19.5 kbp. A single contiguous DNA sequence could not be assembled; this hypothesis is based on consideration of long contigs from each of *A. spiralis* clones PhF-5 and PhF-6, along with manual correction of sequences. **(b)** *A. spiralis* PhF-6 gRNA-encoding maxicircles, also linear, are typically ~ 600 bp in length, and consist of a highly conserved region, along with a single gRNA-encoding region. A subset of 100 contigs was selected for display. **(c)** *P. ankaliazontas* encodes mitochondrial proteins on several *‘*maxicircles’, most of which are circular-mapping. Each maxicircle has a conserved region. **(d)** *P. ankaliazontas* minicircles are also circular-mapping, are typically 1300–2200 bp, encode 2 gRNAs, and contain a conserved region. A subset of 100 contigs were selected for display. Black shading in genes in (**a**) and (**c**) correspond to a gene region that is edited; white regions are unedited. The frequency of U indel editing is presented for each gene in Supplementary Material, Table S2. Shading in (**b**) and (**d**) correspond to sequence identify of ≥ 90%, corresponding to minicircle backbone sequence, whereas divergent are similar to mitochondrial mRNAs, suggesting a role in RNA-editing.

We used mature mitochondrial mRNA sequences to identify mtDNA contigs that specify protein- and rRNA-coding genes (maxicircles) and guide RNAs (minicircles), and to investigate RNA-editing in *P. ankaliazontas* and *A. spiralis* (Fig. 8). Maxicircle contigs were identified for all *P. ankaliazontas* mitochondrial mRNAs, and were characterized by high coverage (> 100 fold higher than nuclear genes), but the assembled sequences differed based on the program used for assembly, with Ray producing longer contigs than SPAdes, ranging from ~ 2–13.6 kbp (SPAdes contigs were not considered further for *P. ankaliazontas*). A given maxicircle contig produced by Ray encodes 1–4 proteins, and contains a region that is ~ 80% identical to portions of other maxicircle contigs. To investigate whether *P. ankaliazontas* maxicircles might be circular-mapping, contigs were assembled using a seed-and-extend approach with NOVOPlasty. Although the resulting contigs were not identical to Ray contigs, they similarly recovered the conserved region of maxicircles, but typically with one protein-coding gene per maxicircle, and most contigs could be circularized. Based on the ability to assemble circular-mapping contigs, we suggest that *P. ankaliazontas* mitochondrial proteins may be encoded on a collection of circular molecules; however, the precise structure of maxicircles remains uncertain and circularization could be an artifact resulting from NOVOPlasty overlapping highly similar regions of the conserved maxicircle backbone. Similar to *P. ankaliazontas*, maxicircle contigs could be identified for all putative *A. spiralis* mitochondrial mRNAs and rRNAs. Low sequence coverage of maxicircles (~ 1–sixfold nuclear genome coverage) from both *A. spiralis* strains PhF-5 and PhF-6 meant that they could not be assembled unambiguously, and was insufficient for seed-and-extend approaches. However, based on comparison of the highly similar PhF-5 and PhF-6 individual assemblies, and manual linkage of assembled contigs using raw reads, *A. spiralis* mtDNA is likely linear, and composed of one or several distinct DNA molecules.

Contigs encoding probable guide RNAs (gRNA) were identified in both *P. ankaliazontas* and *A. spiralis* assemblies. *P. ankaliazontas* gRNA contigs are characterized by high coverage, being ~ 1300–2200 bp in length, and each having conserved regions similar to other gRNA contigs, which are distinct from the conserved region for maxicircle contigs. Most *P. ankaliazontas* gRNA contigs likely encode 2–3 gRNAs, and those examined here could typically be circularized with seed-and-extend approaches. *A. spiralis* gRNA contigs are also characterized by higher coverage, are ~ 600 bp long, and are highly similar to other gRNA contigs, except for a single gRNA-encoding region of ~ 85 bp. *A. spiralis* gDNA contigs could not be circularized using a seed-and-extend approach, and are likely linear molecules. Confirmation of gRNA gene size and number would benefit from RNA-seq of libraries enriched for small RNAs^42^.

Mitochondrial RNA-editing has been described in a wide variety of kinetoplastids, and is dominated by guide-RNA directed uridine insertion/deletion. We found that the magnitude of U indel RNA-editing differs substantially between *P. ankaliazontas* and *A. spiralis*. With the exception of putative rRNA genes, and Cox1, which is edited predominantly in its 5ʹ and 3 ʹ regions, *P. ankaliazontas* mitochondrial mRNAs are characterized by extensive U indel editing along the majority of their length. On average, *P. ankaliazontas* mRNAs have 389 U insertions (range 166–885) and 29 U deletions (range 13–49) per transcript, and the final transcripts are 76% longer than the corresponding gene sequences (range 45–105%). To the best of our knowledge, mitochondrial mRNAs from *P. ankaliazontas* are the most extensively edited reported thus far. In at least 8 of the protein-coding genes, the stop codon was generated by U insertion/deletion. Editing of *A*. *spiralis* mitochondrial transcripts is more concentrated in 5ʹ and 3ʹ regions, although Nad1, Nad8, Nad9, and Atp6 are heavily edited. U insertions average 166 (range 19–331), and deletions average 43 (range 17–80) per transcript, and transcripts are on average 27% longer than gene sequences. In some cases, however, incomplete mRNAs limited our ability to fully reconstruct some editing events. In contrast to mitochondrial mRNAs, putative rRNAs are edited much less extensively (Supplementary Material, Fig. S3). The partial *P. ankaliazontas* 9S rRNA is unedited, and the 12S rRNA sequence indicates no U indel editing. However, there are 12 presumed base deamination edits (8 cytosine-to-uracil and 4 adenosine-to-inosine), observed as C-to-T and A-to-G changes, over a span of 30 nucleotides in the 12S sequence. The presumed deaminative base edits represent the only deaminative edits encountered in our analyses of mitochondrial genes (Supplementary Material, Fig. S4). The putative *A. spiralis* 9S and 12S rRNAs appear to be unedited.

Bioinformatic searches for RNA-editing machinery in *P. ankaliazontas* and *A. spiralis* using *Trypanosoma brucei* homologs as queries reveal a similar repertoire to that found in *Perkinsela*^43^ (Supplementary Material, Fig. S5).

## Discussion

### Evolution of morphology and ultrastructure

Morphological analyses of *P. ankaliazontas* and *A. spiralis* highlight their considerable and detailed similarity to one another, and their utility in reconstructing the ancestral characteristics of kinetoplatids. Specific similarities between the two species include two non-adherent heterodynamic flagella with paraflagellar rods, an apical cytostome, a deep flagellar pocket, terminal and basal plates in the flagellar transitional zone, a flagellar root system and reinforced cytopharynx, systems of subpellicular microtubules, trichocysts, crystalloid structures, and mitochondria containing discoid cristae and small kinetoplasts. *P. ankaliazontas* and *A. spiralis* are both fast-moving eukaryovorous predators that retain prey in large food vacuoles. The morphological differences between these new prokinetoplastids lie in their cell coverings (*e.g.*, glycocalyx *versus* scales), the presence of a skewed groove in *P. ankaliazontas*, but spiral folds in *A. spiralis* on the cell surface, microtubular prism arrangement, the presence of thin hairs on *P. ankaliazontas* flagella, and the absence of a contractile vacuole in *P. ankaliazontas*.

*Papus ankaliazontas* and *A. spiralis* are also similar to other flagellates from freshwater (*Rhynchobodo armata, Pseudophyllomitus apiculatus*)*,* and marine (*Hemistasia phaeocysticola* and *H. amylophagus*) environments^44–47^. This primarily concerns the external morphology of the cells with two heterodynamic flagella, cell coverings reinforced by a system of subpellicular microtubules, a flagellar root system and reinforced cytopharynx, and arrangement of microtubular bands. But some differences are noted in the number of microtubules in the nemadesm; there is a group of several microtubules instead of microtubular prism (nemadesm) in *H. phaecysticola.* Paraflagellar rods and extrusive organelles of *P. ankaliazontas* and *A. spiralis* are similar to their counterparts in the anaerobic flagellate, *Postgaardi mariagerensis* (Symbiontida, Euglenozoa), which contains a hydrogenosome-like organelle^48^.

*Papus ankaliazontas* and *A. spiralis* do not possess pronounced compact kinetoplasts in their mitochondria, like the majority of bacteriovorous bodonids do, but retain some condensations of filamentous osmiophilic material. Such condensations (which we take as homologous to kinetoplasts) were also revealed in *Rhynchobodo armata*, *Pseudophyllomitus apiculatus*, and *Hemistasia phaecysticola*, whereas *H. amylophagus* possesses a large vermiform mitochondrion with kinetoplasts occupying the mid-portion. In typical bodonids (e.g. *Bodo*, *Parabodo*) the kinetoplast is localized near flagellar basal bodies^49,50^, which is not the case in *P. ankaliazontas* or *A. spiralis*.

The structure of the trichocysts from the genera *Papus*, *Apiculatamorpha, Pseudophyllomitus*, *Rhynchobodo, Hemistasia*, *Postgaardi* is quite similar, especially the structure of the discharged extrusome envelopes. The trichocyst coats consist of crossed thin fibrils in these organisms. Similar trichocysts are also found in the bacteriotrophic marine neobodonid *Klosteria bodomorphis*^51^.

*Papus ankaliazontas* and *A. spiralis* also share some similarity with free-living bacterivorous bodonids, most notably the presence of a permanent tubular cytopharynx reinforced with approximately the same set of mircotubules^51–53^^.^ and nemadesms in many cases^54–56^. Nemadesms of *P. ankaliazontas* and *A. spiralis* are probably homologous to the band of 20 microtubules in *Bodo saltans* and *Parabodo caudatus*^49^ as well as the band of 5 microtubules in *Cephalothamnium cyclopum*^57^.

Some ultrastructural features, like a reinforced cytopharynx, the flagellar root system and structure of flagella, and extrusomes also unite *P. ankaliazontas* and *A. spiralis* with heterotrophic diplonemids and euglenids. Pronounced similarity is observed between the trichocysts of *P. ankaliazontas* and *A. spiralis* and the diplonemid *Diplonema nigricans* and heterotrophic euglenid *Entosiphon sulcatum*^58,59^. Extrusive organelles in these species have a tubular envelope with a mesh structure and contain amorphous material subdivided into 4 sections. Several larger trichocysts were also found in the heterotrophic euglenid, *Peranema trichophorum*^60^. A predatory life style, the presence of discharged trichocysts in the digestive vacuoles, and the proximity of the mature trichocysts to the cytopharynx indicate the involvement of these organelles in prey acquisition.

Upon comparison of *P. ankaliazontas* and *A. spiralis* with their closest studied relatives *Ichthyobodo*^61^ and *Perkinsela*^14^, it is clear that certain similarities are observed only with *Ichthyobodo*. This is likely due to the intracellular habitat of the latter species and, as a consequence, the reduction of some of its organelles. *Ichthyobodo* cells possess two flagella, subpellicular microtubules, a reduced cytostomal apparatus (a small number of microtubular bands), and a large number of kinetoplasts. In contrast, *Perkinsela* is characterized by the absence of flagella and basal bodies, a pronounced reduction of most cytoskeleton structures, one or two nuclei, a large kinetoplast, and pellicular microtubules. There is no cytostome, cytopharynx, or microtubular band system. Both *Ichthyobodo* and *Perkinsela* lack trichocysts.

In agreement with a recent multigene analysis based on transcriptome data^16^, phylogenetic reconstructions of the 18S rRNA gene confirm that the eukaryovorous predators, *P. ankaliazontas* and *A. spiralis*, are prokinetoplastids. Given that the only other described prokinetoplastids, *Ichthyobodo* spp. and *Perkinsela* spp., are ectoparasites and endosymbionts, respectively, *P. ankaliazontas* and *A. spiralis* represent the first described free-living prokinetoplastids. However, despite their pronounced morphological and behavioural similarities (see above), these new species are not particularly close relatives of each other within Prokinetoplastina; their 18S rRNA genes are only 89% identical, and they fall with two different clades within the tree.

Overall, *P. ankaliazontas* and *A. spiralis* are most similar morphologically to species from genera outside the prokinetoplastids: *Hemistasia* and *Pseudophyllomitus* (*Phyllomitus*), and especially to *H. amylophagus* and *P. apiculatus* (by external morphology). Molecular data are not available for these or most other con-generic species, and there is considerable taxonomic confusion regarding these organisms. *Hemistasia amilophagus* and *Pseudophyllomitus apiculatus* were originally described as representatives of the genus *Phyllomitus* (*P. amilophagus* Klebs, 1982 and *P. apiculatus* Skuja 1948). Until recently, this genus has also included *P. undulans* Stein 1878, *P. granulatus* Larsen et Patterson, 1990, *P. salinus* Lackey, 1940, *P. vesiculosus* Larsen et Patterson, 1990, *P. yorkeensis* Ruinen, 1938. *Phyllomitus yorkeensis* was later transferred to *Palustrimonas* due to the presence of two opposed flagella, which insert subapically and close together in separate grooves^62^. However, recent analyses based on 18S rRNA gene data demonstrate that this species is one of the basal lineages of Alveolata^63^. The first described *Phyllomitus* (*P. undulans*) is quite different morphologically from subsequently described species, and is characterized by a very distinctive feature: the adherence of the two flagella to each other. Flagella in all other species of *Phyllomitus* are not adhered. Due to this inconsistency, Won Jee Lee erected a new genus, *Pseudophyllomitus*^64^, to accommodate the taxa without adherent flagella, and made 4 new combinations (*Pseudophyllomitus apiculatus*, *Pseudophyllomitus granulatus*, *Pseudophyllomitus salinus*, *Pseudophyllomitus vesiculosus*). He also transferred *Phyllomitus amylophagus* to *Hemistasia amilophagus* based on the similarity with *Hemistasia phaeocysticola* in cell shape, length, flexibility, and the presence of a spiral groove^64^. These two flagellates are very difficult to distinguish by light-microscopy, although they have ultrastructural differences, like an absence of nemadesm, the presence of a distinct glycocalyx-like coat, cysts or cyst-like bodies, and swollen peripheral lacunae in the cytoplasm of *H. phaeocysticola*^45,47^. It was difficult to ascertain, however, whether these ultrastructural differences are of interspecific or intergeneric level.

Furthermore, the first 18S rRNA gene from the representative of the genus *Pseudophyllomitus* (*P. vesiculosus*) revealed that this species is actually not a euglenozoan at all, and is in fact a stramenopile, falling within the clade of previously environmental clones, MAST-6^65^. Furthermore, based on morphology, and following Cavalier-Smith^8^, we believe that the type species of *Phyllomitus* (*P. undulans*) could be a cercozoan (Imbricatea, Marimonadida). Cavalier-Smith^8^ has also proposed that transferring *Phyllomitus amylophagus* to *Hemistasia*^64^ was wrong if Mylnikov et al.^47^ correctly identified their strain, since it lacks cortical alveoli or a well-developed set of encircling microtubules; this species is probably a neobodonid, deserving a new genus. In contrast, other authors have suggested that *Phyllomitus apiculatus*, ultrastructurally studied by Mylnikov^46^, was misidentified and probably belongs to the neobodonid genus, *Rhynchobodo*^8,66–68^ or the diplonemid genus, *Hemistasia*^45^.

Thus, *Phyllomitus* and *Pseudophyllomitus* are not related to each other, and *Pseudophyllomitus* is a polyphyletic genus that unites flagellates from different eukaryotic supergroups, namely stramenopiles and euglenozoans at a minimum. Despite the ultrastructural similarities of *P. ankaliazontas* and *A. spiralis* to some of these flagellates, we cannot use any of the already employed taxonomic names to characterize them: they don’t belong to *Hemistasia* or *Rhynchobodo* based on molecular evidence; they don’t belong to *Phyllomitus* based on morphological evidence (non-adherent flagella); and *Pseudophyllomitus* corresponds to MAST-6 stramenopiles (family Pseudophyllomitidae Shiratori et al. 2017 in Sagenista). Thus, we erect two new genera for these evolutionarily important flagellates.

### Evolution of genomic characteristics

Among kinetoplastids, nuclear genomic data are available for a wide range of parasitic trypanosomatids^69^, a single bodonid, *Bodo saltans*^70^, and the highly reduced endosymbiotic prokinetoplastid*, Perkinsela*^15^. Despite their significant differences at the cellular and ecological levels, kinetoplastid genomes studied thus far typically share a number of derived traits, including a relatively small size and high gene density, a small number of canonical spliceosomal introns, spliced-leader trans splicing, and polycistronic transcription of gene clusters. Genome assemblies for *P. ankaliazontas* and *A. spiralis* largely fit with and extend these trends^71^, revealing these are ancestral states for the kinetoplastida as a whole.

Nuclear genome sizes of kinetoplastids range from 9.5 Mbp in *Perkinsela* to 39.9 Mbp in *Bodo saltans*. *P. ankaliazontas* and *A. spiralis* genome assemblies are highly fragmented, but fell within this range after prey decontamination, at 22.5 and 24.3 Mbp, respectively. However, both assemblies are incomplete, as only 73% and 37% of *P. ankaliazontas* and *A. spiralis* genes (predicted from transcriptome analyses) could be mapped to their respective genomes. Low coverage of both nuclear genomes is a consequence of the very complex nature of the samples, which include co-cultured bacteria and prey kinetoplastids. In fact, even in the case of *P. ankaliazontas*, which was enriched for target-cell DNA via cell-sorting after nearly complete depletion of *P. sorokini* prey, only 44% of read pairs mapped to the decontaminated assembly. This indicates that the complete nuclear genomes of *P. ankaliazontas* and *A. spiralis* are likely considerably larger than those found in other kinetoplastids, and that significant enrichment would be required for lower-throughput, long-read technologies to improve genome assemblies. We attempted to predict the size of the *P. ankaliazontas* and *A. spiralis* nuclear genomes with a two kmer frequency-based approaches^28,72^, using reads that map to the decontaminated nuclear genome assemblies. One approach, GCE, yielded estimated genome sizes of 46 and 70 Mbp for *P. ankaliazontas* and *A. spiralis*, respectively, whereas the bbmap approach could not estimate a reasonable *A. spiralis* genome size (less than 1 Mbp), likely due to low coverage of reads that map to the decontaminated genome, but suggested a genome of 54 Mbp in *P. ankaliazontas*. The kmer frequency-based estimates are consistent with genome size predictions based on transcript mapping rate and decontaminated genome size for *A. spiralis* (37% of transcripts mapping and 24.3 Mbp of assembled sequence), but, based on higher transcript mapping rates and small intergenic distances (below), we suggest that this approach may yield an artificially high value for *P. ankaliazontas*. Unfortunately, *P. ankaliazontas* died after several years in the laboratory, so no further sequencing can be done to test this.

Canonical *cis* spliceosomal introns could not be identified in *P. ankaliazontas* or *A. spiralis*, although the paucity of introns detected may be exaggerated by the highly fragmented nature of the genome assemblies. Introns are exceedingly rare in other kinetoplastids as well, with two described in *T. brucei*^73^*,* and none in either *B. saltans*^73^ or *Perkinsela*, altogether suggesting that intron poverty is also a general and ancestral feature of kinetoplastids. This is in contrast to euglenids and diplonemids, which contain an abundance of canonical and non-canonical introns^74–77^. In contrast to *cis* introns, *trans-*spliced introns are abundant in *P. ankaliazontas* and *A. spiralis*, as they are in all kinetoplastids surveyed to date. The identical SL sequences of *P. ankaliazontas* and *A. spiralis* are distinct from those of other kinetoplastids, which has been an important means of differentiating prokinetoplastid transcripts from those of their bodonid prey.

Gene clustering analysis was limited here by the fragmented nature of the assemblies, but it is clear that biased polarity and gene clustering are characteristics of *P. ankaliazontas* and *A. spiralis* genomes, as in other kinetoplastids. Intergenic distances were very small on average for *P. ankaliazontas* (78 bp), and approximately 1.2 kbp for *A. spiralis. Perkinsela* intergenic distances average 515 bp, whereas they are ~ 1 kbp in *Trypanosoma*. Together, these data indicate that prokinetoplastid nuclear genomes are similar in form to those of better studied kinetoplastids in terms of their coding density and lack of introns, and that differences in basic genome characteristics do not explain their slightly increased size in *P. ankaliazontas* and *A. spiralis*. Rather, we suggest that increased gene number may offer a better explanation, as transcriptome analyses have demonstrated that *P. ankaliazontas* and *A. spiralis* have a more complex metabolic repertoire than other kinetoplastids, and are on par with more versatile free-living euglenozoans such as euglenids and diplonemids^16^.

### Evolution of the mitochondrial genome

Trypanosomatid mitochondrial genomes are made up of two distinct classes of catenated circular DNA molecules: protein- and rRNA-specifying maxicircles (~ 25 kbp), and minicircles, which encode RNAs that serve as a template for RNA-editing reactions. Typically, trypanosomatid mtDNA encodes 18 proteins, including components of respiratory complexes I, III, IV, and V, the mitoribosome, and two reduced ribosomal RNAs, the 9S and 12S subunits^78^. Less is known about the complement of mtDNA-encoded proteins encoded in other kinetoplastid groups. The mitochondrial genome (*i.e.*, maxicircles) of the bodonid, *Bodo saltans*, has been reported as two contigs totaling 25 kbp, encoding 11 proteins, and a fragment of the 12S rRNA gene^79^. The mtDNA of *Perkinsela*—the only prokinetoplastid examined to date—specifies only 6 proteins^43^, a number that reflects the absence of a functional respiratory Complex I (CI; NADH dehydrogenase), and no rRNA genes could be identified. We identified 14 and 12 proteins encoded in the mtDNA of *P. ankaliazontas* and *A. spiralis*, respectively, all of which are also encoded by trypanosomatid mtDNAs. Among these protein-coding genes are CI proteins Nad1, Nad4, Nad5, Nad7, Nad8, and Nad9, demonstrating that loss of CI is not a general feature of prokinetoplastids, and may represent a *Perkinsela*-specific reduction. Proteins encoded in trypanosomatid mtDNA, but not identified in prokinetoplastids, are generally ‘orphans’, and cysteine-rich proteins, which are not highly conserved. Mitochondrial rRNA genes are exceptionally divergent in kinetoplastids, and have proven difficult to identify bioinformatically, though they must exist to support translation of mtDNA-encoded proteins. We found evidence for 9S and 12S rRNA homologs expressed from mtDNA contigs in each of *P. ankaliazontas* and *A. spiralis* (Fig. 7), and identified a candidate 12S transcript in *Perkinsela*^43^. However, prokinetoplastid 9S/12S candidates appear to be truncated, and key domains, including a peptidyltransferase motif of the 12S gene could not be unambiguously identified, suggesting highly divergent and possibly fragmented rRNAs in prokinetoplastids.

The topology of mtDNA differs considerably across kinetoplastids^80^; for instance, maxicircles are circular in *Trypanosoma* with a single ~ 17kbp coding region^80^, but *Perkinsela* maxicircles assemble into three linear contigs capped with telomere-like sequences^43^. We were unable to assemble definitive maxicircle contigs in either *P. ankaliazontas* or *A. spiralis*. Maxicircle sequences were characterized by low coverage in *A. spiralis* PhF-5 and PhF-6, indicating that they do not have the unusually high mtDNA copy number described in *Perkinsela* and diplonemids^81^. We suggest that the *A. spiralis* maxicircle is linear, and is represented by a single contig that is approximately 19.5 kbp. But this suggestion is based mainly on an inability to find reads that could link contig ends into a circle in manual searches, and thus remains tentative. In contrast, *P. ankaliazontas* maxicircles had a higher coverage than in *A. spiralis* (though still not high like *Perkinsela*), but they are complex, and consistent assemblies were not recovered using different assembly algorithms. However, all assemblies suggest that *P. ankaliazontas* mtDNA contains multiple maxicircles, each with a moderately conserved non-coding region, and one to several genes.

Various forms of RNA-editing have been described across eukaryotes^82^; however, gRNA-mediated uridine insertion/deletion editing has only been described in the mitochondria of kinetoplastids and diplonemids^83^, although editing in the latter group is also characterized by *trans*-splicing of split sub-genic modules, along with C-to-U and A-to-I editing. RNA-editing has been characterized extensively in trypanosomatids^78,84^ and some bodonids^85^. The extent of editing observed differs across species and by genes within a species; some mRNAs are unedited, whereas others are edited predominantly in specific subgenic (*e.g.*, 5ʹ and/or 3ʹ) regions, or across their entire length (*i.e.*, pan-editing). Given that mitochondrial genome structure and RNA-editing differ in diplonemids, there has been interest in examining gene expression in the mitochondria of deep-branching kinetoplastids to better understand when trypanosomatid-style RNA editing arose in evolution, and what the ancestral condition was in kinetoplastids. This is especially important because preliminary reports of mtDNA structure from the most basal (uncultured) kinetoplastids using single-cell genomics uncovered tantalizing but preliminary evidence of diplonemid-like properties^86^, with genes split into subgenic modules suggestive of *trans*-splicing. RNA-editing was previously investigated in *Perkinsela*, revealing abundant U indel editing (and misediting), essentially the same as found in bodonids and trypanosomatids^43^. We found that *P. ankaliazontas* and *A. spiralis* mitochondrial RNAs also undergo extensive U indel editing. This is especially pronounced in *P. ankaliazontas*: most mRNAs are pan-edited, with some edited transcripts being more than twice as long as the corresponding gene (Cox3), or with exceptionally high numbers of U insertions (Nad5: 885 insertions; Supplementary Material, Table S2). RNA-editing in *A. spiralis* was less dramatic, resembling editing in *Perkinsela*^43^, with a combination of pan-editing and editing restricted to 5ʹ or 5ʹ and 3ʹ regions. Despite differences in the degree of RNA-editing, the conservation of editing machinery is very similar across prokinetoplastids, suggesting that RNA-editing in *Perkinsela* was not reduced dramatically in the process of becoming an obligate endosymbiont, in contrast to its other cellular and genomic characteristics^15^. The only non-U indel editing observed in our data was a cluster of presumed C-to-U and A-to-I substitutions in the *P. ankaliazontas* 12S rRNA (Supplementary Material, Fig. S4). In mitochondria, the only other report of C-to-U and A-to-I editing of rRNAs is found in diplonemid mRNA and rRNA, where the edits also occur in clusters^87^. This finding therefore constitutes the first evidence that deaminative mitochondrial RNA-editing was present in the common ancestor of diplonemids and kinetoplastids. We found no evidence for mitochondrial *trans*-splicing in any of the identified *P. ankaliazontas* or *A. spiralis* RNAs. This is consistent with other kinetoplastids, but is in contrast to diplonemids^88^, which stitch together mature RNAs from > 20 subgenic modules^89^, and possibly uncultured basal kinetoplastids^86^, which were suggested to have diplonemid-like RNA-editing based on the identification of fissioned mitochondrial coding sequences. Altogether, prokinetoplastid RNA-editing is highly similar to RNA-editing in other kinetoplastids, with the exception of limited C-to-U and A-to-I editing, and it will be important to culture and sequence even more basal kinetoplastids to fully understand the evolution of RNA-editing within Kinetoplastea and Euglenozoa.

### Taxonomic considerations

*Papus* n. gen. Tikhonenkov, Gawryluk, Mylnikov, and Keeling.

#### Assignment

Eukaryota; Discoba; Euglenozoa; Kinetoplastea; Prokinetoplastina.

#### Diagnosis

Elongated heterotrophic flagellates with apical cytostome on the top of the rostrum and two heterodynamic flagella with paraflagellar rods, which originate almost in parallel from the bottom of the deep flagellar pocket. The pronounced groove starts ventrally from the flagellar pocket and turns up to the dorsal side of the cell. Anterior flagellum bears thin mastigonemes. Cell covered with dense, thick layer of structured glycocalyx. Nemadesm consists of four closely situated rows of microtubules (5 + 4 + 3 + 1 arrangement) anteriorly and of two rows (5 + 4 arrangement) close to the posterior cell end. Trichocysts cylindrical in a cross section with osmiophilic envelope and inner cylinder.

#### Etymology

From Persian پاپوش (*pâpuš*, ‘slipper’). Shape of the cell with rostrum resembles pointy toed shoes of Sultan in Turkey, where the protist was found.

#### Zoobank registration

urn:lsid:zoobank.org:act: 349C5006-484D-464D-B140-2E4029158D01.

#### Type species

*Papus ankaliazontas.*

*Papus ankaliazontas* n. sp. Tikhonenkov, Gawryluk, Mylnikov, and Keeling.

#### Diagnosis

Rigid, elongated cylindrical cell, 15–20 μm long, with roundish posterior end and slightly asymmetrically located anterior rostrum. Anterior flagellum is slightly shorter than the cell body, posterior flagellum two times longer than the cell. Flagellates swim fast and rotate around their longitudinal axis. The two flagella often wrap spirally around the anterior part of the body, leading the cell to stop. Predator of heterotrophic flagellates. Binary division. Marine.

#### Type material

A block of chemically fixed resin-embedded cells of the type strain, PhM-4, is deposited in the Marine Invertebrate Collection, Beaty Biodiversity Museum, University of British Columbia as MI-PR152. This constitutes the name-bearing type of the new species (a hapantotype).

Figure 2a illustrates a live cell of strain PhM-4.

#### Type locality

Macrophyte-associated detritus at the shore of a brackish lagoon (~ 8‰) named Lake Küçükçekmece (Istanbul, Turkey).

#### Etymology

From Greek *αγκαλιάζοντας*, *ankaliázontas* (embracing). Two flagella often wrap spirally around the anterior part of the body.

#### Gene sequence

The 18S rRNA gene sequence has the accession number GenBank: MW346654.

Zoobank Registration. urn:lsid:zoobank.org:act:DF6CBBCF-3EA1-4363-8C1B-79CBBEF46F9C.

*Apiculatamorpha* n. gen. Tikhonenkov, Gawryluk, Mylnikov, and Keeling.

#### Assignment

Eukaryota; Discoba; Euglenozoa; Kinetoplastea; Prokinetoplastina.

#### Diagnosis

Heterotrophic flagellates with apical cytostome on the top of pronounced rostrum and two heterodynamic flagella with paraflagellar rods, covered with a fine fiber layer, originate almost in parallel from the bottom of the deep flagellar pocket. Cells covered with layer of spherical and lamellate scales and possess spiral folds across the cell body surface. Nemadesm consists of three closely situated rows of microtubules (7 + 8 + 7 arrangement). Contractile vacuole lies near flagellar pocket. Irregular rows of trichocysts lie at the anterior part of the cell.

#### Etymology

From Latin ‘*apiculata*’, adjective inflection of ‘*apiculātus’* (pointed, abrupt) and ‘*morpha’*(‘-shaped’).

#### Zoobank registration

urn:lsid:zoobank.org:act: CC149041-D89D-448C-8432-64E9492602D3.

#### Type species

*Apiculatamorpha spiralis.*

*Apiculatamorpha spiralis* n. sp. Tikhonenkov, Gawryluk, Mylnikov, and Keeling.

#### Diagnosis

Elongate oval, elongate oviform or pear-shaped cell, 7–13.5 μm long. Pronounced rostrum located anteriorly, posterior cell end is roundish. Anterior flagellum is about cell length, posterior flagellum two times longer than the cell. Both flagella often wrap spirally around the cell body. Flagellates swim fast and rotate around their longitudinal axis. Predator of heterotrophic flagellates. Binary division. Freshwater.

#### Type material

A block of chemically fixed resin-embedded cells of the type strain, PhF-6, is deposited in the Marine Invertebrate Collection, Beaty Biodiversity Museum, University of British Columbia as MI-PR153. This constitutes the name-bearing type of the new species (a hapantotype).

Figure 4a illustrates a live cell of strain PhF-6.

#### Type locality

Macrophyte-associated detritus in a small freshwater lake near Noi Bai International Airport (Hanoi, Vietnam).

#### Etymology

Latin ‘*spiralis*’ (spiraling). Cell possesses spiral folds across the cell body surface.

#### Gene sequence

The 18S rRNA gene sequence has the accession number GenBank: MW346645.

#### Zoobank registration

Urn:lsid:zoobank.org:act: 2D248DB2-FC83-4155-B1F2-BC42E51541E4.

## Conclusion

Here we characterized the first free-living prokinetoplastids, which are represented by small predatory eukaryovorous flagellates. Taking the diverse geographic locations where the strains were isolated into account (Turkey, Indonesia, and Vietnam), and the sources of closely related environmental sequences (a deep sea hydrothermal field in the North Atlantic for *P. ankaliazontas*, and freshwaters of Panama and Botswana for *A. spiralis*), it appears that free-living prokinetoplastids are cosmopolitan but, as eukaryovorous predators, are probably not very abundant, as they occupy the upper levels of microbial food webs. Notably, recent metagenomic investigations show that kinetoplastids make up a very small proportion of protists among marine plankton, and that the previously known prokinetoplastid genera, *Ichthyobodo* and *Perkinsela*, are virtually undetectable in plankton, perhaps owing to their associations with fish^90–92^. Our analyses (not shown) also fail to detect close relatives of *P. ankaliazaontas* or *A. spiralis* in marine metagenomic datasets. This indicates that predatory prokinetoplastids may be rare, or predominantly associated with sediments. Alternatively, ‘universal primers’ may not amplify the V9 hypervariable region of prokinetoplastids efficiently; to this end, the universal reverse V9 primer has two mismatches relative to *P. ankaliazontas* and *A. spiralis* 18S genes. It will be important to obtain behavioural, ecological, and genomic data from more free-living prokinetoplastids in order to improve our understanding of the ancestral ecological roles of the group (*i.e.* are most species eukaryovores?), and how parasitic and endosymbiotic life histories evolved.

The phylogenetic position, genome structure, morphology, and lifestyle of these newly described protists fill in important knowledge gaps regarding the ancestral state of kinetoplastids as a whole^71^, and the changes in morphology and molecular traits leading up to the origin of the parasitic and endosymbiotic life histories of the best-studied species in the group. Free-living prokinetoplastids possess relatively large nuclear genomes that are likely intermediate in size between diplonemids and other kinteoplastids, although further work is necessary to determine genome size more accurately. Similarly, free-living kinetoplastids have more complex metabolic repertoires than other kinetoplastids, and are more similar to versatile free-living euglenozoans such as euglenids and diplonemids in this sense^16^. Our analysis also suggests that intron poverty is a general feature of kinetoplastids, in contrast to diplonemids and euglenids, which contain an abundance of canonical and non-canonical introns.

*Papus ankaliazontas* and *A. spiralis* mitochondrial RNAs undergo extensive U indel editing, which is especially pronounced in *P. ankaliazontas,* where most mRNAs are pan-edited with high numbers of U insertions. This is similar to RNA-editing in kinetoplastids, but the identification of putative deaminative base editing in *P. ankaliazontas*, otherwise seen only in diplonemids, suggests that the widespread loss of C-to-U and A-to-I editing in kinetoplastid mitochondria. Nonetheless, the RNA-editing machinery is very similar across parasitic and free-living prokinetoplastids, as well as other kinetoplastids, altogether suggesting that the machinery required for U indel editing was already well-developed in the ancestor of kinetoplastids. However, our interpretation of all these characters—as well as the larger question in kinetoplastid evolution on the origin of parasitism—may be significantly altered once we have further information about prokinetoplastid diversity and the currently uncharacterized basal kinetoplastids. These organisms are only known from environmental clones, but to fully understand the evolution not only of genomic but also behavioural and ecological characters, acquiring more data from these organisms is critical.

## Supplementary Information

Supplementary Information.

## Data Availability

The datasets generated and analyzed during the current study are available at the NCBI BioProject PRJNA549754 (Sequence Read Archive accession numbers SRX9813564–SRX9813567).
