# Supplementary material for: First finding of free-living representatives of Prokinetoplastina and their nuclear and mitochondrial genomes
Source: Sci Rep. 2021 Feb 3;11:2946. doi: 10.1038/s41598-021-82369-z (PMC7859406; doi:10.1038/s41598-021-82369-z)
Supplement: Supplementary file 1 — Supplementary Information. [file 41598_2021_82369_MOESM1_ESM.pdf]

Scientific Reports—Article

Supplementary Information

First finding of free-living representatives of Prokinetoplastina and their nuclear and mitochondrial genomes

Denis V. Tikhonenkov[1,2,*,#], Ryan M. R. Gawryluk[3,#], Alexander P. Mylnikov[1,†], Patrick J. Keeling[4]

[1] Papanin Institute for Biology of Inland Waters, Russian Academy of Sciences, Borok, 152742, Russia

[2] AquaBioSafe laboratory, University of Tyumen, 625003, Tyumen, Russia

[3] Department of Biology, University of Victoria, Victoria, V8W 2Y2, British Columbia, Canada.

[4] Department of Botany, University of British Columbia, Vancouver, V6T 1Z4, British Columbia, Canada

[†] Deceased May 30, 2019

[*] Correspondence: tikho-denis@yandex.ru

# equal contribution

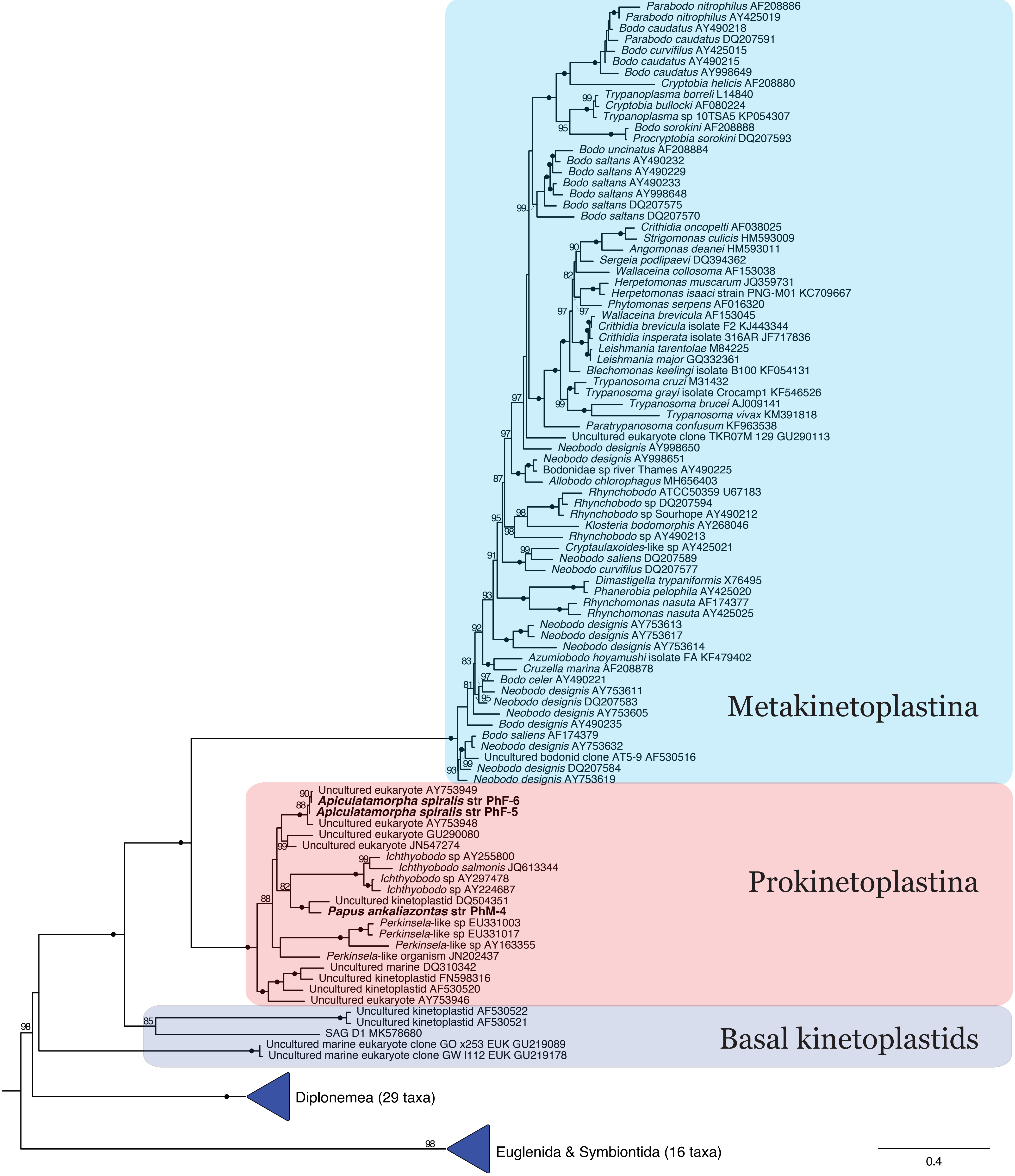

Supplemental Figure S1. A maximum likelihood phylogeny of the 18S ribosomal RNA gene from diverse kinetoplastids, diplonemids, and euglenozoans (outgroup). Reconstructed with IQ-TREE v1.6.12 using the GTR+F+I+G4 model with 200 initial parsimony trees and 1000 ultrafast bootstrap replicates as a measure of support.

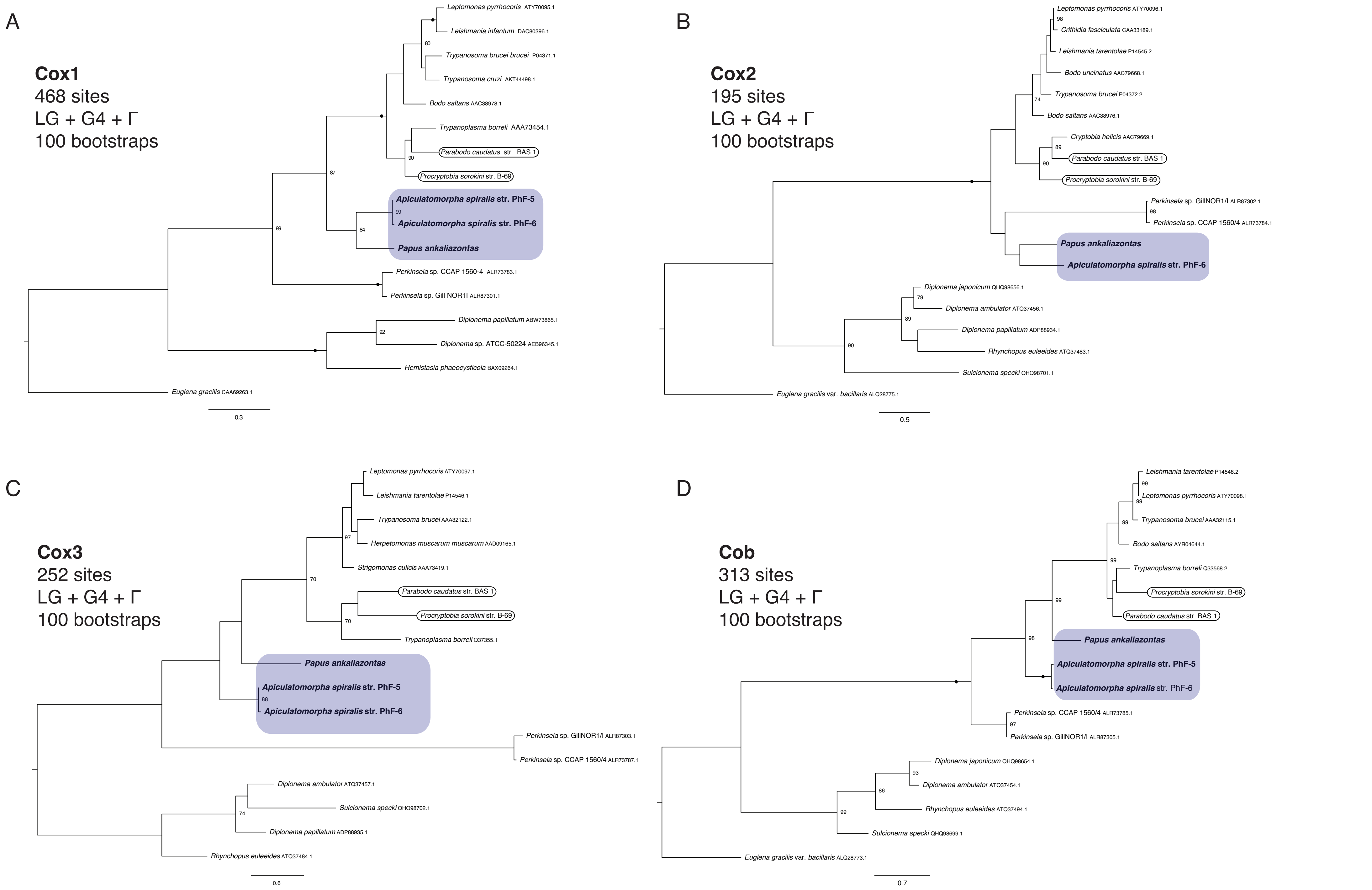

Supplementary Figure S2. Maximum likelihood phylogenies of mtDNA-encoded proteins demonstrate that they do not derive from prey bodonids. Phylogenies of (a) cytochrome c oxidase subunits I (Cox1), (b) II (Cox2), (c) III (Cox3), and (d) cytochrome b (Cob) are shown. The prokinetoplastids *P. ankaliazontas* and *A. spiralis* are indicated by bolded text inside of a coloured box; the names of their prey are enclosed in boxes. Non-parametric bootstrap values are show as measures of statistical support; for clarity, values less than 70 are not shown and values of 100 are represented by black circles.

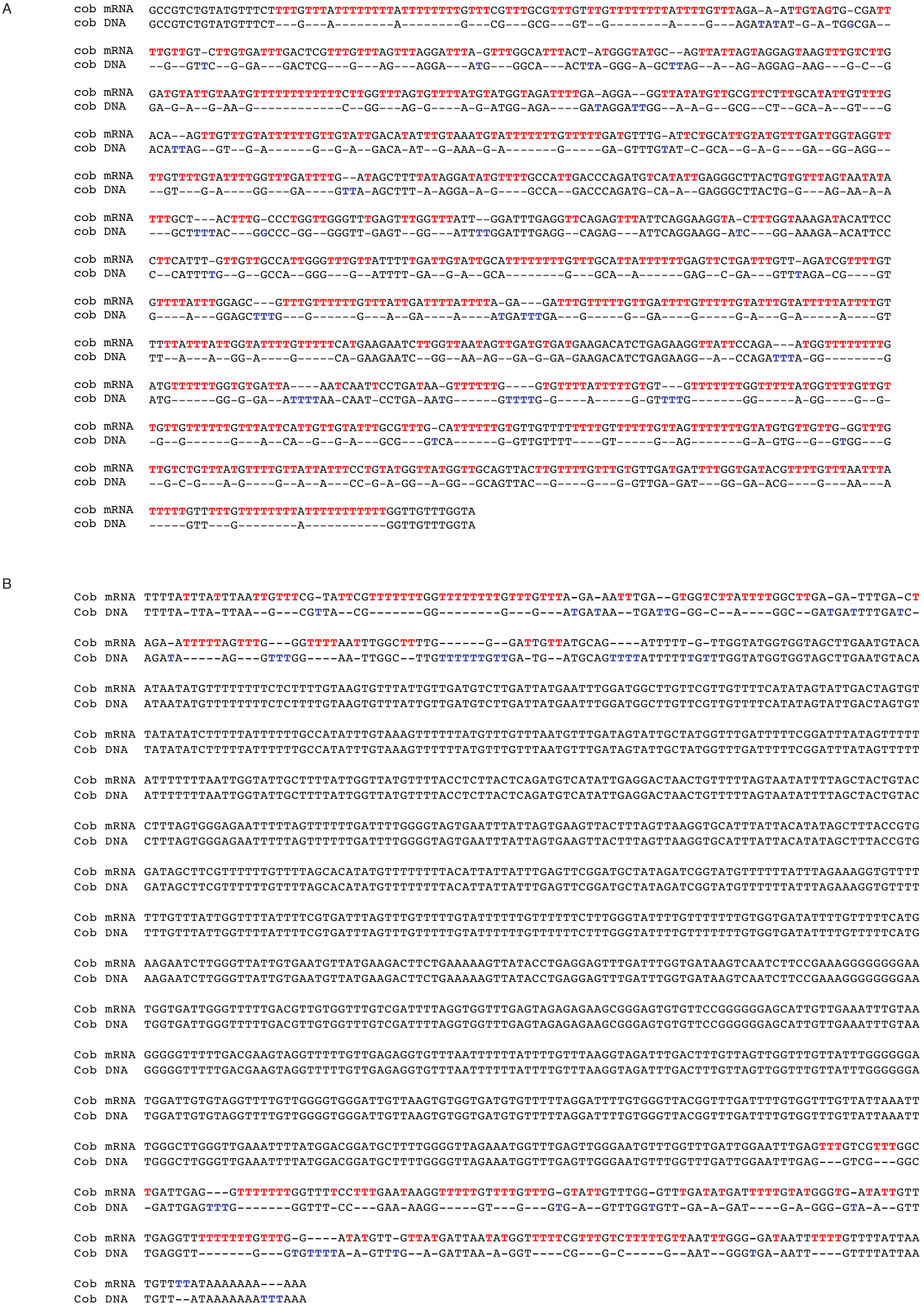

Supplementary Figure S3. Representative U indel editing in *Papus ankaliazontas* and *Apiculatamorpha spiralis*. Nucleotide alignments of the cytochrome b gene and edited mRNA sequences are presented for (a) *P. ankaliazontas* and (b) *A. spiralis*. Editing of Cob illustrates the broadscale patterns of RNA-editing observed in these two new species: *P. ankaliazontas* mitochondrial mRNA transcripts are extensively edited along their entire length, whereas *A. spiralis* counterparts were typically edited at their termini. Bold red nucleotides denote U insertions and blue nucleotides denote U deletions (although represented by T). Cob is the 6th most heavily edited mRNA in each of *P. ankaliazontas* and *A. spiralis*.

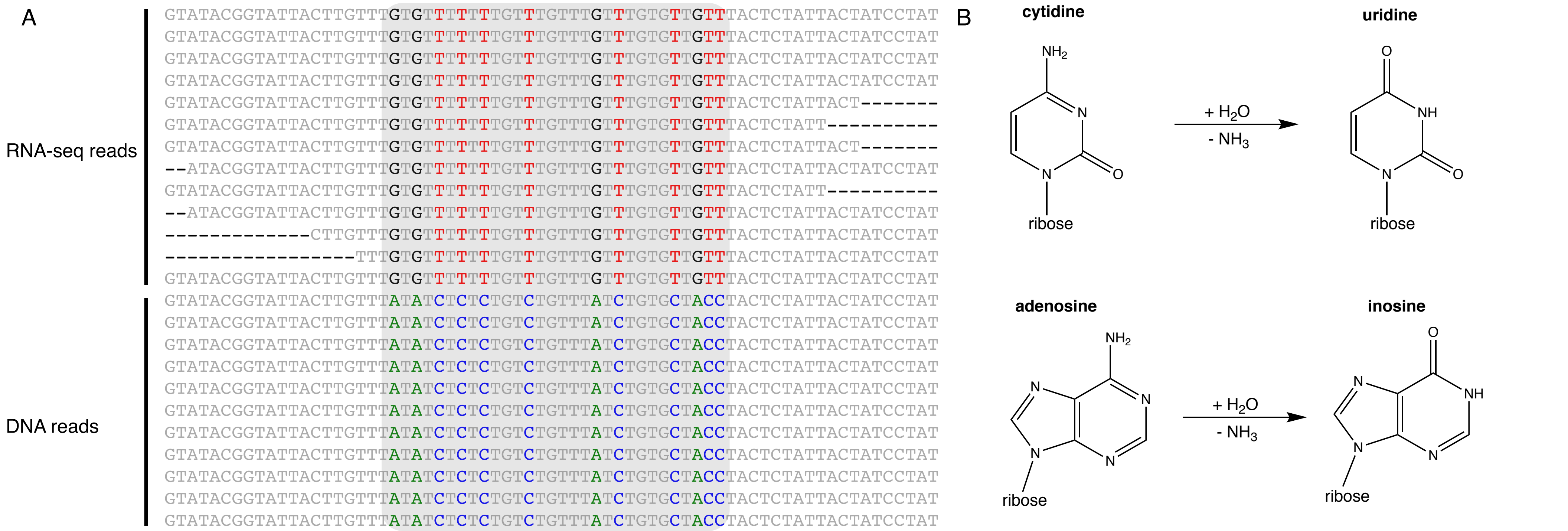

Supplementary Figure S4. Evidence for C-to-U and A-to-I RNA-editing in the putative *Papus ankaliazontas* mitochondrial 12S rRNA. (a) Multiple alignment of RNA-seq and genomic DNA reads from *P. ankaliazontas*. Deaminative editing of cytosine to uracil and adenosine to inosine is observed as C-to-T and A-to-G changes, respectively. A cluster of 12 deamination edits were observed over a short span of the 12S rRNA, representing the first description of deaminative editing in kinetoplastids; no incompletely edited sites were observed. (b) Chemical reactions underlying C-to-U and A-to-I RNA-editing.

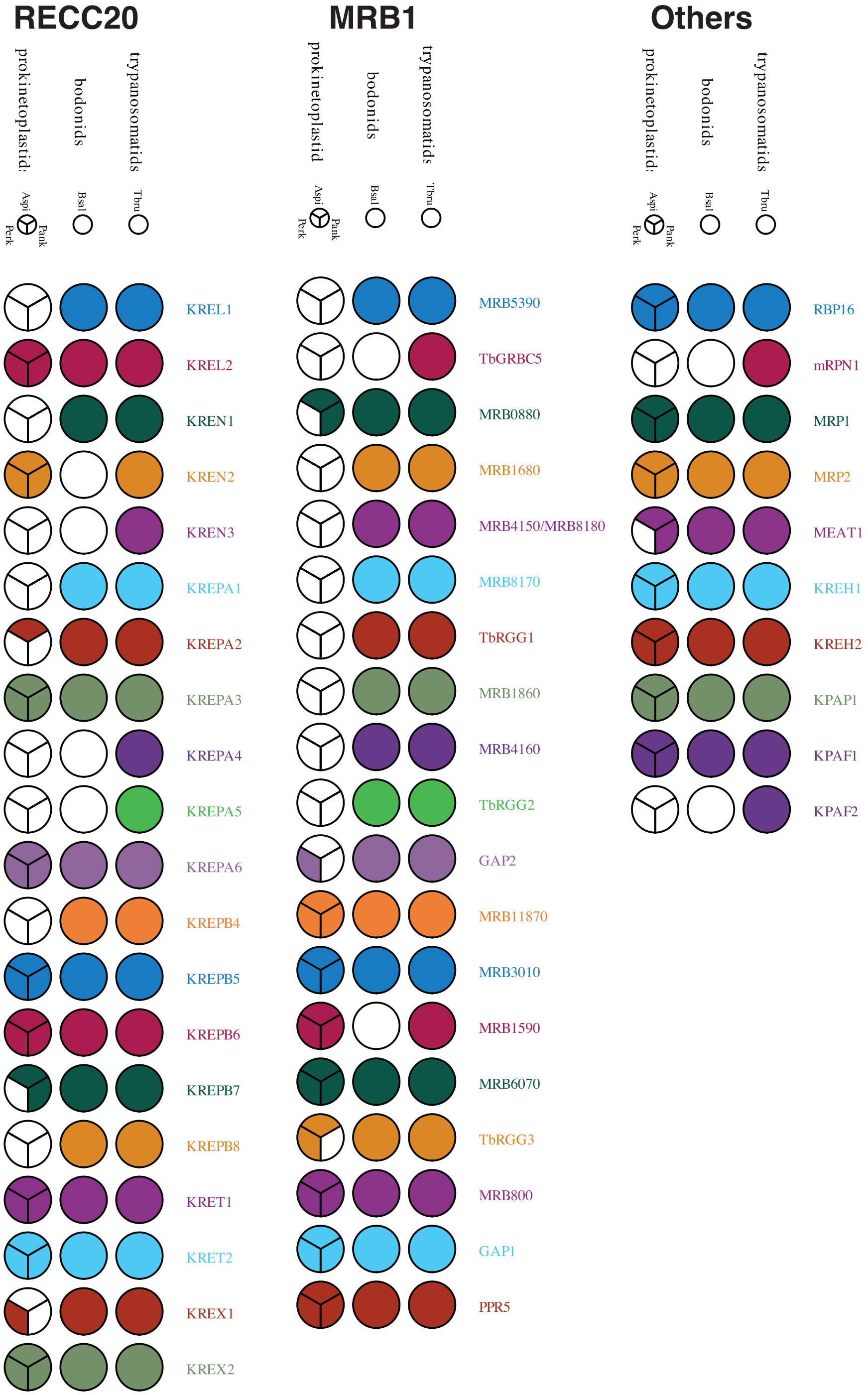

Supplementary Figure S5. RNA-editing machinery in prokinetoplastids. Coulsen plot highlighting the presence and absence of Trypanosoma brucei RNA-editing components in Bodo saltans, and the prokinetoplastids, Perkinsela, Papus ankaliazontas, and Ankaliazontas spiralis PhF-6. To accommodate all genes, the plot shows components from the RECC20 and MRB1 and 'others' separately. Filled in wedges indicate the presence of a subunit, and empty wedges indicate absence.

Supplemental Table S1. Statistics for *Papus ankaliazontas* and *Apiculatomorpha spiralis* genome assemblies. Genomes were assembled for each species with SPAdes, and decontaminated of prey kinetoplastids and co-cultured bacteria using a combination of Autometa (for prokaryotes), and blastn using transcriptome assemblies from cultures that contained the eukaryotic prey. Most statistics are derived from Quast analysis; however, transcript mapping rate and number of introns are derived from BLAT analyses, paired read mapping rate – referring to the percentage of genomic DNA reads that map to the decontaminated genome assemblies – were derived using bbmap, and transcript number refers to number of transcriptome coding sequences predicted by Transdecoder. Predicted genome size corresponds to Bayesian kmer-based estimates generated by GCE. Intergenic distances are based on manually inspected gene mapping on several of the longest contigs.

	P. ankaliazontas	***A. spiralis***
Contigs	5,118	9,927
N50 (bp)	5,464	2,438
Assembly length (bp)	22,454,015	24,339,364
Longest contig (bp)	31,864	25,801
GC (%)	55.8	48.02
Coverage (SPAdes)	6	2
N's per 100 kb	86.59	0.82
Transcript number	21,181	32,854
Transcript mapping rate (%)	73	37
Number of introns	0	0
Intergenic distance (bp)	~78	~1200
Paired read mapping rate (%)	44	6.5

Supplemental Table S2. Table of RNA editing statistics for *Papus ankaliazontas* and *Apiculatomorpha spiralis* strain PhF-6, based on comparisons between edited mitochondrial mRNAs identified in transcriptome datasets and genomic contigs.

Species	Gene	mRNA completeness	Gene completeness	Editing Type	Pre-edited length	Edited ORF Length	% Size increase	Insertions	Deletions	Observations
P. ankaliazontas	Nad1	complete	Uncertain 5'	Pervasive central and 3' editing, diminished at 5' end.	394	684	73,60406091	329	39	Stop codon formed by 3 U deletion.
P. ankaliazontas	Nad4	incomplete	internal problems	Pervasive central and 5' editing, diminished at 3' end.	711	1028	44,58509142	349	32	Cannot assemble a full-length mRNA lacking internal stop codons. Dominant version has stop codons, but is highly supported by the reads. Possible pseudogene? Stop codons exist in a high GC area, suggestive of loss of editing.
P. ankaliazontas	Nad5	complete	complete	pan	805	1517	88,44720497	885	49	Stop codon generated by U insertion. Required some manual curation.
P. ankaliazontas	Nad7	complete	complete	pan	723	1218	68,46473029	542	47	Stop codon generated by U insertion.
P. ankaliazontas	Nad8	complete	complete	pan	274	456	66,42335766	207	25	
P. ankaliazontas	Nad9	incomplete	incomplete	Pervasive central and 3' editing, diminished at 5' end.	265	467	76,22641509	218	16	Cannot assemble full-length mRNA. Truncated at 5' end, without a good candidate start codon. Like Nad4, there is a GC-rich region, supported by reads, that looks like it should be edited, but isn't.
P. ankaliazontas	Cob	complete	complete	pan	642	1195	86,13707165	575	49	2 possible G deletions. Stop codon generated by U insertion.
P. ankaliazontas	Cox1	complete	complete	5' and 3' editing	1392	1665	19,61206897	282	10	U insertion generates start codon. U insertion generates stop codon.
P. ankaliazontas	Cox2	complete	complete	pan	340	651	91,47058824	322	11	Stop codon generated by U insertion.
P. ankaliazontas	Cox3	possibly complete	likely complete	pan	377	771	104,5092838	411	17	Stop codon generated by U insertion. Possibly truncated at 5' end.
P. ankaliazontas	Atp6	incomplete	unknown	pan	349	693	98,56733524	383	39	
P. ankaliazontas	rps12	likely complete	complete	pan	162	315	94,44444444	166	13	Stop codon generated by U insertion. No ATG start identified, but likely complete based on MSA.
P. ankaliazontas	Murf1	incomplete	complete	unknown (likely pan)	162	310	91,35802469	150	1	Cannot resolve 3' end of transcript
P. ankaliazontas	putative 9S	possibly complete	complete	unedited	676	676	0	0	0	
P. ankaliazontas	putatitve 12S	possibly complete	complete	minimal	1050	1050	0	0	0	C-to-U, A-to-I editing
A. spiralis	Nad1	complete	complete	pan	481	732	52,18295218	331	80	
A. spiralis	Nad4	incomplete	complete	5' editing	1874	1899	1,334044824	42	17	Possible pseudogene (N-terminal part of protein in transcript but broken in all contigs)
A. spiralis	Nad5	possibly complete	complete	5' editing	1196	1347	12,62541806	198	47	
A. spiralis	Nad7	complete	complete	5' and 3' editing	1128	1192	5,673758865	96	32	

A. spiralis	Nad8	complete	complete	pan	278	462	66,18705036	236	52	
A. spiralis	Nad9	incomplete	complete	pan	322	525	63,04347826	248	45	Termini incomplete in cDNA, in frame stop
A. spiralis	Cob	incomplete	complete	5' and 3' editing	1373	1479	7,720320466	148	42	Likely more editing at 5' and 3' ends but not helped by transcripts
A. spiralis	Cox1	possibly complete	complete (but two contigs)	5' editing	1476	1476	0	19	19	
A. spiralis	Cox2	complete	complete	5' and 3' editing	519	594	14,45086705	128	51	
A. spiralis	Cox3	complete	complete	5' and 3' editing	762	795	4,330708661	79	46	
A. spiralis	Atp6	possibly complete	complete	pan	206	357	73,30097087	190	39	
A. spiralis	putative 9S	possibly complete	complete	unedited	334	334	0	0	0	
A. spiralis	putatitve 12S	possibly complete	complete	unedited	1108	1108	0	0	0	
